# Advances and Challenges in Targeting TGF-β Isoforms for Therapeutic Intervention of Cancer: A Mechanism-Based Perspective

**DOI:** 10.3390/ph17040533

**Published:** 2024-04-20

**Authors:** David Danielpour

**Affiliations:** 1 Case Comprehensive Cancer Center Research Laboratories, The Division of General Medical Sciences-Oncology, Case Western Reserve University, Cleveland, OH 44106, USA; dxd49@case.edu; Tel.: +1-216-368-5670; Fax: +1-216-368-8919; 2 Department of Pharmacology, Case Western Reserve University, Cleveland, OH 44106, USA; 3 Institute of Urology, University Hospitals, Cleveland, OH 44106, USA

**Keywords:** TGF-β, tumor progression, oncogene, fibrosis, therapeutics

## Abstract

The TGF-β family is a group of 25 kDa secretory cytokines, in mammals consisting of three dimeric isoforms (TGF-βs 1, 2, and 3), each encoded on a separate gene with unique regulatory elements. Each isoform plays unique, diverse, and pivotal roles in cell growth, survival, immune response, and differentiation. However, many researchers in the TGF-β field often mistakenly assume a uniform functionality among all three isoforms. Although TGF-βs are essential for normal development and many cellular and physiological processes, their dysregulated expression contributes significantly to various diseases. Notably, they drive conditions like fibrosis and tumor metastasis/progression. To counter these pathologies, extensive efforts have been directed towards targeting TGF-βs, resulting in the development of a range of TGF-β inhibitors. Despite some clinical success, these agents have yet to reach their full potential in the treatment of cancers. A significant challenge rests in effectively targeting TGF-βs’ pathological functions while preserving their physiological roles. Many existing approaches collectively target all three isoforms, failing to target just the specific deregulated ones. Additionally, most strategies tackle the entire TGF-β signaling pathway instead of focusing on disease-specific components or preferentially targeting tumors. This review gives a unique historical overview of the TGF-β field often missed in other reviews and provides a current landscape of TGF-β research, emphasizing isoform-specific functions and disease implications. The review then delves into ongoing therapeutic strategies in cancer, stressing the need for more tools that target specific isoforms and disease-related pathway components, advocating mechanism-based and refined approaches to enhance the effectiveness of TGF-β-targeted cancer therapies.

## 1. Introduction

Transforming growth factor-betas (TGF-βs) constitute a family of three unique structurally similar multifunctional cytokines in mammals (TGF-βs 1, 2, and 3) which are crucial for regulating various developmental and physiological processes, spanning from embryonic development to tissue maintenance [1,2,3,4]. Importantly, signaling by TGF-βs confers robust tumor suppression in most normal tissues [5]. Of note, this review employs the general term TGF-β to address characteristics likely common to all three isoforms. However, the specific isoform is specified when identified or utilized in a particular study. A dogma in this field is that the signaling pathways downstream of the receptor binding of each of the three TGF-βs isoforms are essentially the same regardless of the isoform triggering those pathways. As such, for simplicity, most investigators in the field refer to TGF-β signaling in their studies rather than specifying the specific isoform used or identified to drive it. However, this could be construed as an oversimplification, often causing investigators to mistakenly assume a uniform functionality among all three TGF-β isoforms.

The dysregulation of TGF-βs can precipitate the pathogenesis of numerous diseases, including cancer and fibrosis [6,7]. In most cancers, the tumor suppressor function of TGF-β signaling is not only lost but instead, through poorly understood mechanisms, TGF-β signaling functionally switches to a driver of tumor growth and progression [8,9]. Such deregulation has incentivized the targeting of TGF-βs and their signaling mediators for therapeutic intervention for cancers.

TGF-βs signal through specifically binding to and promoting the dimerization of transmembrane receptors (TβRI and TβRII), which collaborate to directly phosphorylate and thereby activate the transcription factors Smad2 and Smad3 [10,11]. Both of these Smads cooperate with Smad4 and many other transcription factors/co-regulators to control the expression of a vast array of TGF-β target genes in a cell-type- and tissue-dependent manner. TGF-β receptors also function independently of Smads, particularly in cancer cells, to activate various other signaling pathways through so-called non-canonical TGF-β signaling pathways [12].

Alterations in TGF-β signaling in cancer can manifest across various key levels: (1) the expression and activation of TGF-β ligands, (2) the expression, post-translational modification, and occurrence of inactivating mutations of TGF-β receptors, (3) the expression, post-translational modification and occurrence of inactivating mutations within Smads, (4) perturbations in Smad co-regulators, and (5) the activation of non-canonical TGF-β pathway signaling.

Pharmacological inhibitors targeting aberrant TGF-β signaling have emerged as promising candidates for cancer treatment. Through an examination of preclinical studies and ongoing clinical trials, this review aims to outline the current landscape of TGF-β inhibitors, assessing their efficacy, challenges, and potential synergies with existing cancer therapeutic modalities. However, given the intricate network of TGF-β signaling pathways, ligand distributions, and diverse functions, a comprehensive understanding of its biology is imperative for optimal targeted therapeutic interventions. This review thereby delves into the molecular intricacies of TGF-β ligands, investigating their distributions, regulation of expression, mechanisms of activation, and physiological roles across different cellular contexts. Furthermore, the review scrutinizes the various mechanisms underlying the dysregulation of TGF-βs expression and signaling mediators in cancer. By shedding light on the molecular intricacies governing TGF-β signaling dysregulation, particularly regarding TGF-β isoforms, this review seeks to offer insights into potential therapeutic avenues for mitigating its adverse effects.

At the ligand level, substantial evidence provided in this review collectively supports that TGF-β1 is the most ubiquitously expressed member of its family. Moreover, TGF-β1 is also the most commonly overexpressed TGF-β isoform in cancers, which also uniquely functions as a potent immune suppressor, thereby enabling tumor cells to survive by escaping immune surveillance mechanisms. This suggests that the overexpression of TGF-β1 is the isoform most likely to drive the progression of cancers. As such, selective therapeutic targeting of the TGF-β1 isoform is likely to be most beneficial to cancer patients, particularly in combination with other therapeutics such as immune checkpoint blockade inhibitors. Despite the known selectivity of the TGF-β1 isoform linked to cancer, the vast majority of TGF-β blockade therapeutics to date indiscriminately target all TGF-β isoforms, an effect likely to contribute to the adverse effects of such therapies given the indispensable role of the other isoforms in normal tissues.

As detailed in this review, many TGF-β inhibitors robustly inhibit the growth and metastatic progression of cancers in animal models. An increasing number of these inhibitors have entered phase I and II clinical trials, demonstrating acceptable toxicity profiles and therapeutic potential over the standard of care. In most studies, TGF-β inhibitors have been shown to work most effectively when combined with other therapeutics rather than as signal agents. These combined therapeutic benefits stem from several potential mechanisms, the most common of which is the link between TGF-β signaling and the development of resistance to standard cancer therapeutics.

## 2. Early Times in TGF-β Research

### 2.1. TGF-β Discovery

TGF-β1 stands as the pioneer within a highly evolutionarily conserved family and superfamily of ligands. Following its discovery as a transforming growth factor activity (formerly named TGF for transforming growth factor) in virally transformed NIH-3T3 fibroblast cell conditioned medium [13], TGF chromatographically fractionated into two separate acid-stable activities on a C18 reverse phase HPLC column, as measured by the phenotypic transformation of the NRK-49F non-tumorigenic kidney fibroblast cell line grown in soft agar [14]. The first peak (fractions 25–30) was named TGF-α, and the second peak (fractions 45–47) was named TGF-β. Both activities were first believed to be tumor-specific autocrine growth factors, and intriguingly TGF-α, but not TGF-β, competed for the binding of ^125^I-EGF to specific, saturable high-affinity binding sites on cells known as the EGF receptor (EGFR) [14,15], which also binds to heparin-binding EGF-like growth factor (HB-EGF), betacellulin, amphiregulin, and epiregulin [16].

At first, TGF-α and TGF-β were believed to be made and secreted specifically by cancer cells rather than normal cells, and cancer cells, through a viral transformation-specific mechanism elaborated such potent, unique transforming activities. However, this hypothesis quickly lost favor following their identification in normal tissues [15,17,18,19,20,21,22], although tumor cells were known to secrete far higher levels of these factors than normal cells. Another radical change in thinking came about following the characterization of TGF-β as a very potent inhibitor of cell proliferation [23,24,25,26]. In contrast to its currently accepted pleiomorphic nature, some early findings generated on its growth inhibitory activity were instead dismissed as contamination in TGF-β preparations. Only after multiple failed attempts to remove such potential contamination and further independent investigations was it clear that TGF-β was also a potent inhibitor of cell proliferation [23]. Thereafter, researchers identified that the mode of TGF-β’s effects depended on cell type and context. For example, while TGF-β stimulated the proliferation and anchorage-independent growth of NRK-49F cells in the presence of EGF [15], TGF-β inhibited the ability of EGF to induce non-malignant rat 3T3 fibroblasts transformed by Myc (Myc-1 cells) to form large colonies in soft agar. In contrast, in the presence of platelet-derived growth factor (PDGF), TGF-β stimulated Myc-1 cells to form colonies in soft agar, while in monolayer culture the same concentrations of TGF-β inhibited the PDGF-induced proliferation of Myc-1 cells [23]. Moreover, TGF-β was shown to robustly inhibit the anchorage-independent growth (in soft agar in the presence of serum) of a variety of cancer cell lines, including A-549, Calu-6, A-373, A-2058, B16F1, MCF-7, and HT-1020 [23]. 

### 2.2. Discovering the Tumor Suppressor and Oncogenic Functions of TGF-β

In 1987, compelling in vivo evidence highlighting the growth inhibitory role of TGF-β emerged. This pivotal discovery involved the strategic placement of pellets infused with TGF-β1 within the developing mouse mammary gland [27]. Furthermore, corroborative findings extended beyond the mammary gland, encompassing various tissues and transgenic mice engineered to overexpress TGF-β1 [28,29,30]. TGF-β’s function as a tumor suppressor appeared in 1995 through the identification of TGF-β receptor mutations in human colon carcinoma with microsatellite instability [31], and the tumor suppressive function of TGF-β was further tested in mouse studies [32,33]. Various studies showed that the loss of TGF-β receptor function is a common feature of many cancers [34,35,36], that an enforced inactivation of TGF-β receptor signaling alone can endow non-tumorigenic cells with the full capability of forming tumors [37], and that the restoration of TGF-β receptor signaling confers tumor suppression [35,38]. Thus, the accepted paradigm was that TGF-β was a potent tumor suppressor in many tissues, making TGF-β a misnomer. With time, the field made a final 180-degree turn in re-establishing its function as a tumor promoter. Subsequent research revealed both tumor-suppressive and tumor-promoting effects of TGF-β on carcinoma cells and in the context of the tumor microenvironment (TME) [8,39,40]. 

### 2.3. Identification of TGF-β Isoforms

TGF-β was renamed TGF-β1 following the identification and isolation of other TGF-β isoforms from various sources. The second identified TGF-β isoform was named TGF-β2 [41,42,43] and was shown to have a more limited expression pattern than TGF-β1 [44,45,46]. Owing to its yet more limited and unique tissue and cell type expression pattern [45,47,48,49,50], TGF-β3 was the third family member to be discovered following its cDNA cloning [47,51]. 

Early research in the TGF-β field focused on the expression, regulation, and activation of the various TGF-β isoforms. However, before a full understanding of the unique regulation and function of each TGF-β isoform, many researchers in the field switched their course of investigation from TGF-β ligands to TGF-β signaling mediators following the isolation and characterization of TGF-β receptors and their signaling mediators, thereby leaving a void in the continuity of research on TGF-β ligands. 

### 2.4. The TGF-β Superfamily

TGF-βs share approximately 30–40% sequence homology with other functionally distinct protein groups encompassed within the larger TGF-β superfamily. This superfamily comprises over 33 members, which include inhibins, activins, bone morphogenetic proteins (BMPs), growth differentiation factor (GDF), Mullerian inhibiting substance (MIS), Leftys, Nodal, Neurturin, Persephin, and others [52,53,54]. While downstream pathways mediate the biological responses of various families in this superfamily, much research supports a significant amount of crosstalk and common signaling mediators. Readers are referred to several thorough reviews on this superfamily for more detail [5,10,55].

## 3. TGF-β Ligands, Their Function, Expression, and Regulation

Today, a significant portion of investigators engaged in TGF-β research tend to erroneously assume a uniform functionality among all of its three isoforms. Consequently, there is a prevailing inclination to concentrate predominantly on studying TGF-β1, often disregarding potential disparities between the isoforms as insignificant. To illustrate, a cursory examination of PubMed reveals a mere 696 and 433 titles that explicitly include TGF-β2 and TGF-β3, respectively. In stark contrast, a similar search on TGF-β1 and TGF-β identifies over 9935 and 22,479 publications, respectively, underscoring the prevailing emphasis on TGF-β1.

TGF-β isoforms are characterized by a conserved arrangement of nine cysteine amino acids required for their relatively preserved shared tertiary structure but share only 71% to 76% amino acid sequence homologies [42,51]. Notably, an exceptional feature of each TGF-β isoform is its remarkably evolutionarily preserved amino acid (aa) sequence, with a near-perfect amino acid sequence identity between distant relatives such as humans and chickens; two-way BLAST alignment of the mature sequence (last 112 aa) of TGF-β2 in *Homo sapiens* (Accession# NM_001135599.4) with that of *Gallus gallus* (Accession# NM_001031045.4) is 99.1% identical, with only one conserved substitution. This evolutionary conservation suggests each isoform plays a critical non-redundant function and that a small alteration in their primary sequence is incompatible with survival. In line with this, TGF-β1 homozygote null knockout mice either die from yolk sac defects during embryogenesis or die within one month of birth from autoimmunity [56,57]. Both TGF-β2 null mice and TGF-β3 null mice die perinatally, with the TGF-β2 null mice having various craniofacial, skeletal, retinal, renal, and heart defects, while TGF-β3 null mice manifest cleft palate and delayed lung development [58,59,60]. The variations in observable traits among mice without a functional gene align with disparities in how tissues express specific genes. For instance, TGF-β1 is found in various stages of development and adulthood, reflecting the prevalent multifocal inflammatory ailment observed in TGF-β1 null mice. On the other hand, TGF-β2 and TGF-β3 exhibit substantial expression in the lungs and heart during development, mirroring significant developmental abnormalities in these organs among the respective null mice.

The most significant disparities among TGF-β isoforms manifest at the expression level, with abundant evidence illustrating distinct spatial and temporal expression patterns of both mRNAs and proteins in developing tissues, regenerating tissues, and pathological responses [1]. Each of the TGF-β isoforms is encoded on a different gene, is located on a different chromosome, and has a unique set of gene promoter and enhancer elements [61]. At the protein level, TGF-β1 is the most widely expressed isoform in this family [45,46,50]. Various immune cells express high levels of TGF-β1 but limited to no TGF-β2 and TGF-β3 [62,63]. This is consistent with far higher levels of TGF-β1 than TGF-βs 2 and 3 found in the spleen [45,50]. Interestingly, although TGF-β1 and TGF-β2 are expressed by a variety of different types of cells, TGF-β3 appears to be expressed mainly by mesenchymal cells [47] and is mainly found in the mammary gland, spleen [45], and placenta [50]. Conditional knockout studies revealed that the expression of TGF-β1 in immune cells regulates immune cell function and that TGF-β3 plays both overlapping and differential immunological roles with TGF-β1 [62]. Furthermore, there is limited evidence that endogenous TGF-β2 has immunosuppressive functions or is involved in the evasion of tumor immune surveillance [46]. 

**Figure 1:**
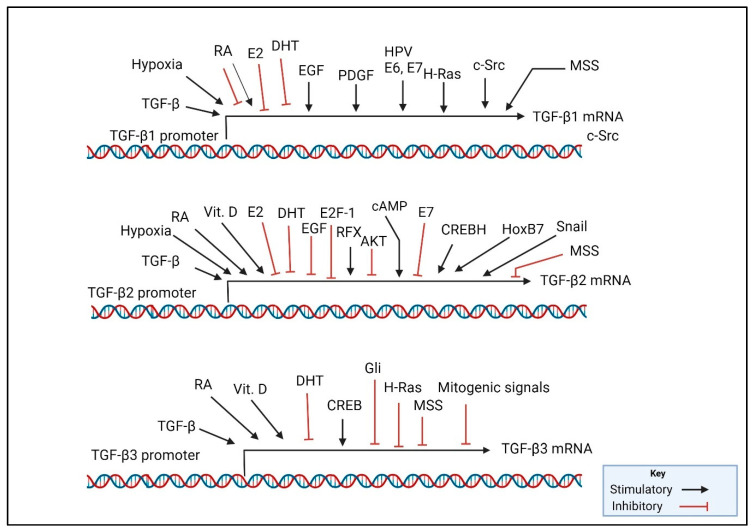
**Regulators of the transcriptional expression of TGF-βs 1, 2, and 3.** TGF-β1 emerges as the predominant isoform upregulated in tumors, correlating with increased cell proliferation activity and malignant transformation. Inducers of proliferation typically induce the expression of TGF-β1 expression while inhibiting the expression of TGF-β2 and TGF-β3. Conversely, conditions promoting growth arrest and differentiation typically selectively induce the expression of TGF-βs 2 and 3 over that of TGF-β1. Abbreviations: AKT (Akt/PKB serine-threonine kinase), RA (retinoic acid), ATF2 (activating transcription factor 2), CREB-1 (cAMP-responsive element binding protein-1), CREBH (cAMP-responsive element-binding hepatocyte protein), DHT (dihydrotestosterone), E2 (estradiol), HoxB7 (Homeobox B7 protein), RFX (regulatory factor x), MSS (mechanical sheer stress), and VD (1,25-dihydroxyvitamin D_3_). These data provide potential triggers for isoform induction in cancer, offering opportunities for TGF-β isoform-targeted therapeutic approaches.

Importantly, the TGF-β1 isoform of this family is the member that is most upregulated in cancers and thus most tightly linked to the oncogenic functions of TGF-β. For example, the malignant transformation of mammary cells is accompanied by an elevation of TGF-β1 at the protein level but a reciprocal loss of TGF-β3 [45]. This is not just a coincidence. A study using mRNA expression from The Cancer Genome Atlas highlighted TGF-β1 as the most common isoform in various human tumors [64]. There is a correlation between the mitotic rate/proliferation activity of tissues and TGF-β1 levels. In broad terms, the expression of TGF-β1 tends to increase in response to signals that encourage cell growth and division, while the expression of TGF-βs 2 and 3 is prompted by signals associated with cellular differentiation and growth inhibition. For instance, growth-stimulatory molecules, like EGF and H-Ras, usually boost the production of TGF-β1 and may inhibit levels of TGF-β2 (Figure 1) [21,65,66,67,68]. On the other hand, serum withdrawal, or treatment with substances that promote growth arrest and differentiation, like retinoic acid (RA), dexamethasone (Dex), cAMP, and 1, 25-dihydroxyvitamin D_3_ (VD), tend to favor the induced levels of TGF-βs 2 and 3 over that of TGF-β1 or even suppress TGF-β1 (in the case of RA and Dex) [65,68,69,70,71,72]. PDGF stimulates the expression of TGF-β1 in NRK-49F fibroblasts [73]; however, its impact on other TGF-β isoforms remains unknown. Aside from proliferative signaling, mechanical shear stress (MSS) force also upregulates the expression and activation of TGF-β1 while it downregulates the expression of TGF-βs 2 and 3 [74]. On the other hand, estradiol (E2) and dihydrotestosterone (DHT), which stimulate growth, suppress the expression of both TGF-βs 1 and 2 in ER+ breast cancer cells and prostate epithelial cells, respectively [75,76].

The elevation of TGF-β2 expression under growth suppressive conditions may be explained in part by the activity of the retinoblastoma protein Rb. In scenarios where signals suppress growth, there is usually an increase in the activity of Rb. This increase facilitates the interaction of ATF2 (activating transcription factor 2) with the TGF-β2 promoter, which subsequently leads to the activation of TGF-β2 expression [77]. The binding of ATF2 to the TGF-β promoter provides part of the mechanistic basis for the induced expression of TGF-β2 by RA [78,79]. Elevated TGF-β2 expression under growth arrest conditions is also controlled through the transcriptional induction of TGF-β2 by the regulatory factor x (RFX), an inhibitor of cell proliferation. RFX is downregulated in cancers, thus driving the suppression of TGF-β2 expression [80]. Through various mechanisms, many, if not most, cancers exhibit an enhanced activation of the survival factor of Akt/PKB. The activation of Akt suppresses the TGF-β2 promoter through phosphorylating the forkhead factor FKHRL1, thereby preventing the transcriptional activation of the TGF-β2 promoter by FKHRL1 [81]. The papillomavirus 16, which is involved in the etiology of some cancers, represses the TGF-β2 promoter in NIH-3T3 cells through an interaction with the HPV E7 oncogene to a promoter region spanning −528 to −251 [82]. In contrast, HPV E6 and E7 induce the expression of TGF-β1 promoter in cervical cancer [83]. In line with mitogen signaling, the overexpression of E2F-1 also similarly suppressed TGF-β2 promoter activity [82].

Despite these mechanisms, in certain cancerous tissues, TGF-β2 is found to be elevated through incompletely unexplored mechanisms. One potential mechanism for the enhanced expression of TGF-β2 in certain cancers may be through the autoinduction of TGF-βs, which serves as a mechanism of signal amplification; each of the three TGF-β isoforms significantly enhances the expression of all three isoforms [84]. It is thus likely that the elevated expression of TGF-β2 in certain cancers is driven in part by an autoinduction of enhanced TGF-β1 signaling. Another mechanism of TGF-β2 overexpression in cancer is tumor hypoxia, which also drives tumor aggressiveness and therapeutic resistance [85,86]. Hypoxia activates the TGF-β2 promoter at a region spanning −77 to −40 [86]. Elevated TGF-β2 in certain cancers may also involve the expression of the transcription factor Snail, which was shown to selectively upregulate the TGF-β2 isoform in pancreatic acinar cells in a pancreatic cancer (PC) model [87]. The transcription factor ATF3, which is associated with endothelial-to-mesenchymal transformation, selectively binds the TGF-β2 promoter, also driving TGF-β2 expression [88]. The proto-oncogene c-Src induces the expression of both TGF-βs 1 and 2, as shown in lens epithelial cells [89]. Furthermore, the Homeobox B7 protein (HoxB7) transcription factor, which is upregulated in breast cancer, drives the selective transcription of TGF-β2 [90]. Important in hepatic fibrogenesis by the hepatitis C virus (HPV) is the induction of TGF-β2 expression mediated by the binding of the cAMP-responsive element-binding hepatocyte protein (CREBH) to the TGF-β2 promoter [91]. Also notably, TGF-β2 stands out as the predominant isoform in body fluids like amniotic fluid, breast milk, and the aqueous and vitreous humor of the eye [1], suggesting that ductal cells are wired to induce the expression of TGF-β2. Carcinomas resulting from those ductal cells (i.e., ductal carcinomas) may thus also be inherently programmed to elevate TGF-β2 expression. 

The pool of identified TGF-β3 regulators is comparatively small. Through an exploration of Gene Expression Omnibus profiles, the author pinpointed several key regulators. Notably, in hepatic stellate cells, the expression of TGF-β3 is robustly induced by cAMP-responsive element binding protein-1 (CREB-1) [92]. Additionally, H-Ras oncogene transformation of fibroblast robustly suppresses TGF-β3 mRNA (Gene Expression Omnibus; profile GDS1801/U03491). Likewise, the transformation of kidney epithelial cells with the Gli oncogene completely suppresses the expression of TGF-β3 mRNA (Gene Expression Omnibus; profile GDS3550/1367859). Despite the dearth of data, these profiles collectively suggest that potent oncogenes can markedly suppress TGF-β3 expression. Moreover, there has not been a clear induced expression of TGF-β3 in cancer, as reported by various cancer studies [93]. Instead, some evidence supports the anti-cancer role of this isoform.

In addition to their differential regulation, each of the three TGF-β isoforms has certain other unique functions. For example, TGF-β1 and TGF-β3 but not TGF-β2 impede the growth of large vessel endothelial cells [1]. Conversely, TGF-β2 and TGF-β3 but not TGF-β1 hinder the survival of cultured embryonic chick ciliary ganglionic neurons. Impressively, TGF-β3 stands out as the isoform with antifibrotic function and inhibits scar formation following injury, which contrasts with the profibrotic activity of the other TGF-β isoforms [94]. Tissue explant studies have further supported distinctions between isoforms. For example, treatment with exogenous TGF-β3 but not with TGF-β1 or TGF-β2 reprograms TGF-β3 null embryos to undergo normal palate development [95,96,97]. Additionally, gene replacement studies, involving the “knock-in” of mature TGF-β1 into the TGF-β3 locus, and vice versa, resulted in a partial, though incomplete, phenotypic rescue of the mutant allele [98,99]. These findings underscore the inherent variations between isoforms that influence their distinct in vivo functions. 

It is worth highlighting that TGF-β2 is also unique among its isoforms due to the absence of an RGD integrin-binding sequence in its precursor [1], a sequence that is necessary for the activation of latent TGF-β1 and TGF-β3 by integrins, as will be described later in this review. Another unique feature of TGF-β2 compared to the other isoforms, as will be discussed in more detail later, is the dependence of β-glycan for TGF-β2 receptor cell signaling. Moreover, the activity of TGF-β2 is >10-fold suppressed over that of TGF-β1 by the abundant plasma protein α2-macroglobulin (α2M), supporting that on a molar basis, TGF-β2 is less active than TGF-β1 in interstitial spaces and that TGF-β2 plays a more localized role (autocrine, paracrine) than does TGF-β1 [100]. In addition, human platelets, which degranulate and release their contents in tumor tissues, are an abundant source of TGF-β1 but not the other isoforms, as will be covered in great detail later. Collectively, these studies support that TGF-β1 is more likely to drive tumor progression than TGF-β2 or TGF-β3 in cancers. It thus follows that, given the important and non-redundant functions of each TGF-β isoform, isoform-selective antagonists, particularly against TGF-β1, are more likely to be effective and safer cancer therapeutic tools than pan TGF-β inhibitors, particularly in the context of immunotherapy. However, it is prudent to assess the expression of all TGF-β isoforms in each type of tumor or each patient for a more targeted therapeutic approach. 

Notably, most clinically available TGF-β antagonists block all three TGF-β isoforms, likely contributing to dose-limiting cardiotoxicities associated with the nonselective inhibition of multiple TGF-β isoforms [101,102], particularly TGF-βs 2 and 3 [58,103,104], consistent with cardiac syndrome from mutations in TGF-βs 2 or 3 genes [105,106,107,108,109,110]. 

## 4. TGF-β Biosynthesis and Activation

**Figure 2:**
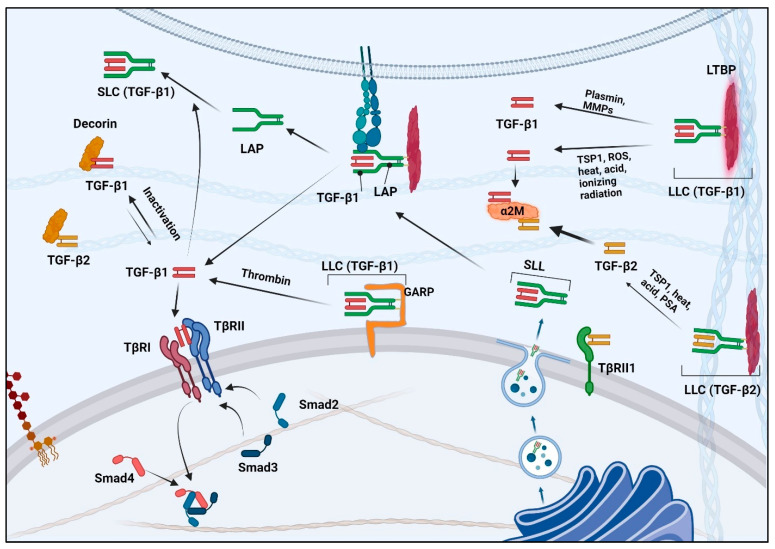
**The intricate processes of TGF-β activation within the intracellular and extracellular environments.** Initially synthesized as homodimers with pro-peptides, mature TGF-βs are cleaved from latency-associated proteins (LAPs) by furin-like enzymes in the trans-Golgi. They are then secreted as small latent complexes (SLCs) bound to LAPs, often associating with latent TGF-β binding proteins (LTBPs) or glycoprotein A repetitions predominant (GARP) to form large latency complexes (LLCs) anchored to the extracellular matrix (ECM), or in the case of GARP, on the surface of specific cells. Activation of TGF-βs can occur via proteolytic cleavage or conformational changes induced by mechanical forces, integrins, reactive oxygen species (ROS), and other effectors. Notably, different isoforms of TGF-β are activated by distinct factors. Once activated, TGF-βs either bind to TGF-β receptors or are sequestered in an inactive form bound to extracellular matrix proteins such as decorin or the plasma protein alpha-2 macroglobulin (α2M), the latter of which has a 10-fold higher affinity for TGF-β2 than TGF-β1. Understanding the complexities of TGF-β activation offers insights into potential therapeutic interventions targeting aberrant TGF-β signaling.

TGF-βs are synthesized as homodimers with a lengthy pro-peptide at the N-terminal region followed by a segment containing a mature TGF-β located at their C-termini [17,111]. Within the trans-Golgi, mature dimeric TGF-βs (25 kDa) are cleaved from their dimeric pro-peptides (known as latency-associated proteins or LAPs) by furin-like enzymes. Mature TGF-βs are secreted from cells as non-covalent, biologically inactive complexes with their LAPs, 75 kDa complexes referred to as small latent complexes (SLCs) (Figure 2) [112]. SLCs frequently associate covalently with a larger latent TGF-β binding protein (LTBP), a family of four distinct members ranging from 125 to 240 kDa [113,114,115]. The association of an SLC with an LTBP comprises what is known as a TGF-β large latency complex (LLC). The LTBP components of LLCs are embedded in extracellular proteins, which in effect anchor latent TGF-βs to the ECM rather than allowing them to diffuse freely in extracellular spaces. Thus, LTBPs are thought to serve as reservoirs for latent TGF-βs in the extracellular environment [116]. The TGF-β1 and TGF-β3 LAP moieties of SLCs are also noncovalently associated with integrins β1, β6, and β8, which contribute to the activation of those TGF-β isoforms.

Once associated with their respective LAP, TGF-βs remain inactive until physiological or pathological processes invoke their activation. There are two main mechanisms for TGF-β activation: activation through proteolytic cleavage and activation through conformational changes induced by extracellular cues [116]. Plasmin or matrix metalloproteinases (MMPs) and kallikreins are typically found to cleave the latent TGF-β complex, promoting the release of mature, active TGF-βs (Figure 2) [116]. These forms of activation are associated with various physiological and pathological processes, including wound healing, fibrosis, and cancer. 

Many non-enzymatic mechanisms underlying the activation of TGF-βs have been identified. For example, mechanical forces such as tension and compression can induce conformational changes in a latent TGF-β complex, freeing an active TGF-β ligand [117,118]. A non-enzymatic activation of TGF-β may occur through interactions with thrombospondin-1 (TSP-1), the integrin αvβ6, reactive oxygen species (ROS) [119], heat, low pH [120,121,122,123], and ionizing radiation (Figure 2) [124]. The glycoprotein A repetitions predominant (GARP) localized on the surface of T cells and platelets also non-enzymatically activates TGF-βs from their latent forms [125,126], with a greater activation of TGF-β1 than TGF-β2 or TGF-β3 [127]. Interestingly, thrombin and other components of the coagulation system seem to be required for the activation of latent TGF-β1 by GARP [127,128]. All of these effectors appear to activate TGF-βs through modifications of LAPs. However, not all TGF-β isoforms are activated by the same factors. For example, integrin αvβ6 activates only TGF-βs 1 and 3 but not TGF-β2 [129], while the prostate-specific antigen (PSA) kallikrein activates only TGF-β2 [130] and ROS activates only TGF-β1 [131]. Importantly, IGF-I which increases the growth and invasiveness of breast cancer cells, stimulates the activation of TGF-β1 [132]. However, IGF-I’s impact on the activation of other TGF-β isoforms remains unknown. Both the enzymatic and non-enzymatic activators of TGF-β1 are commonly associated with tissue injury, inflammation, and cancer [116,133]. 

Given that free LAP can associate with and neutralize TGF-β, TGF-β activation necessitates the capture or physical alteration of LAP. A crystallographic investigation of TGF-β1 LLC has unveiled that when the αvβ6 integrin binds to the LAP portion of that complex, it triggers a modification in the conformation of LAP. This conformational shift results in the release of active TGF-β isoforms 1 and 3 [134]. Intriguingly, the LAP of each TGF-β isoform differs in its efficiency of neutralizing its corresponding mature ligand, with a half-maximal inhibition (IC_50_) of 0.1 nM, 0.62 nM, and 3.0 nM for TGF-βs 1, 2, and 3, respectively, aligning with their relative intrinsic ease of activation [135]. 

The complexities of the mechanism by which TGF-βs are activated allow various tissues to exert fine-tuned spatial-temporal control of TGF-β activity, but this also provides more places where things can go wrong with TGF-β signaling and responses. However, defining defects underlying the mechanism of TGF-β activation offers opportunities for effective targeted therapeutics. 

## 5. TGF-β Binding Proteins

Mature TGF-βs are highly hydrophobic, so much so that stock concentrations need to be maintained at very low pH conditions (e.g., 4 mM HCl) to prevent their rapid binding to plastic. Thus, most of the mature TGF-βs in biological fluids are “sticky” and are hence likely to appear as non-covalent complexes with other proteins. Aside from TGF-β receptors and cell surface binding proteins, mature TGF-βs are tightly, and with high specificity, bound to several plasma and extracellular matrix proteins. Such binding proteins dictate the availability and activity of TGF-βs in tissue compartments and likely play differential roles in their autocrine/paracrine versus endocrine activities. 

Plasma contains exceedingly low concentrations of TGF-β compared to high levels of TGF-β found in serum, supporting that most of the TGF-β in serum is released from platelets following their degranulation [46]. However, most of the TGF-β found in serum is found in a non-covalent latent complex associated with α_2_M [46,136], which differs from the latent form in platelets [137]. α_2_M is a highly abundant 720 kDa plasma protein that binds to and neutralizes many proteases, particularly those activated during hemostasis. α2M also binds to several growth factors in the circulation. Both the LAP and α_2_M complexes of TGF-β are biologically inactive, as they do not bind to TGF-β receptors or stimulate TGF-β responses. α_2_M binds to TGF-β2 with a substantially higher affinity than it does to TGF-β1. α_2_M also effectively blocks the binding of TGF-β2 to TGF-β receptors and selectively inhibits TGF-β2′s biological activity [100,138]. Importantly, TGF-βs bind to the fast or activated form of α_2_M [100], which is the form modified by proteases and also cleared by receptor-mediated endocytosis. This difference in isoform inactivation likely plays a role in the differential activities of these isoforms in tissues as well as their clearance. 

Aside from α_2_M, TGF-βs bind to proteoglycans in the extracellular matrix (ECM). Decorin, a proteoglycan associated with ECM that functions as a tumor suppressor, binds to the mature forms of all three TGF-β isoforms, thereby blocking TGF-β signaling [139,140]. Tumorigenesis and tumor progression are marked by the loss of decorin expression [139], thereby relieving decorin’s inhibitory effect on TGF-βs. Thus, the activation of TGF-β signaling in certain cancers is driven, in part, by the loss of decorin expression. Given decorin’s tumor suppressor function, this proteoglycan is a promising therapeutic target for cancer. Two other proteoglycans found in the ECM also bind to TGF-βs with high affinity [141] and affect the bioavailability of TGF-βs. Together, the modulation of the levels of these proteoglycans in ECM provides potential therapeutic strategies for controlling TGF-βs in tumors and during fibrosis.

## 6. TGF-β Receptors

**Figure 3:**
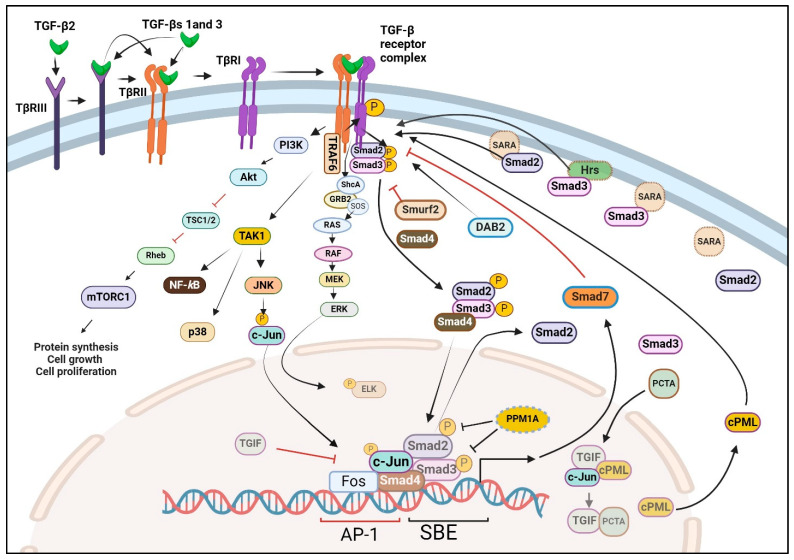
**TGF-β receptor binding and downstream canonical and non-canonical signaling pathways.** Upon encountering TGF-β1, the TGF-β type II receptor (TβRII) prompts a conformational change that allows for the recruitment of the TGF-β type I receptor (TβRI), forming a complex comprising two TβRIIs and two TβRIs. Conversely, TGF-β2 requires TβRIII (also called β-glycan) for cellular responses due to its inability to directly bind TβRII or TβRI. The formation of the TβRII-TβRI-ligand complex triggers the phosphorylation of TβRI by TβRII. This event activates TβRI, leading to the phosphorylation of downstream Smads, particularly Smads 2 and 3 (the canonical pathway). SARA (Smad anchor for receptor activation) and Hrs/Hgr (hepatocyte growth factor-regulated tyrosine kinase substrate) are crucial for the delivery of R-Smad to the TβRII-TβRI complex for R-Smad activation. Additional proteins involved in delivering R-Smad to the TGF-β receptors include DAB2 (Disabled-2) and cPML (cytoplasmic promyelocytic leukemia protein). Normally confined to the nucleus, cPML is sequestered in a tertiary complex with transcription factor c-Jun and the transcriptional repressor TGIF (TG-interacting factor). Upon TGF-β stimulation, PCTA (PML competitor for TGIF association) translocates into the nucleus, where it competes with cPML for TGIF binding. This competition leads to the export of cPML to the cytoplasm, where it interacts with R-Smads, thereby promoting R-Smad-TβRI interaction. After phosphorylation, Smads 2 and 3 form heterotrimeric complexes with Smad4 and translocate into the nucleus, where they regulate the transcription of target genes by interacting with other transcription factors and co-regulators. Meanwhile, inhibitory mechanisms, including the action of Smad7, ubiquitin ligases, and the nuclear phosphatase PPM1A (magnesium-dependent protein phosphatase A1) work in concert to deactivate TGF-β signaling, ensuring its dynamic control. In the non-canonical pathways of TGF-β signaling, various adapters are recruited to the activated TβRI-TβRII complex independent of Smads, triggering various kinase signaling cascades that ultimately promote cell growth, survival, cell migration, and invasion. Other abbreviations: TSC1/2 (tuberous and tuberin sclerosis complexes 1 and 2); JNK (c-Jun N-terminal kinase); c-Jun (cellular Jun transcription factor, subunit of the AP-1 complex); c-Fos (cellular Fos proto-oncogene, AP-1 transcription factor subunit); ELK (E26 transformation-specific (ETS)-like protein); Rheb (Ras homologue enriched in brain).

TGF-β receptors (TβRs) were initially discovered through the cross-linking of [125] I-labeled TGF-β1 to proteins on the cell surface of intact cells (45). This led to the identification of three distinct transmembrane proteoglycans that serve as TGF-β binding proteins across various cells, which include TβRI, TβRII, and TβRIII, ~55 kDa, ~75 Da, and ~280 kDa, respectively [142,143,144]. Upon isolating and cloning the receptor genes [145,146,147], it was revealed that both TβRI and TβRII are transmembrane glycoproteins with an extracellular ligand-binding domain and an intracellular serine-threonine kinase domain [144,148]. Additional cell surface binding proteins that interact with all three TGF-β isoforms, such as TβRIV (60–64 kDa proteoglycan) and TβRV (400 kDa proteoglycan) have also been reported [148,149,150,151]. Whilst TβRI is essential for transmitting TGF-β1 responses, it cannot directly bind to TGF-β1. Instead, TGF-β1 binds to TβRII, inducing a conformational alteration in TβRII that facilitates the recruitment of TβRI. This forms a complex where a single dimeric TGF-β molecule is bound by two TβRIIs and two TβRIs (Figure 3) [152,153,154]. In contrast to TGF-β isoforms 1 and 3, TGF-β2 cannot directly bind to either TβRII or TβRI. In cells expressing endogenous levels of TβRII and TβRI, the cellular responses to TGF-β2 require the involvement of TβRIII (also called β-glycan) (Figure 3). TβRIII is a highly glycosylated transmembrane protein with a short cytoplasmic segment devoid of a kinase segment [155]. Unlike TβRII, TβRIII exhibits relatively high-affinity binding (Kd~0.1 nM) to all three mammalian TGF-β isoforms and can enhance the biological activity of these isoforms by facilitating their interaction with TβRII [156]. Under certain circumstances like inflammation and carcinogenesis, the extracellular segment of TβRIII may detach from cells. Both its soluble and complete versions can also impede TGF-β responses [157,158].

Once the TβRII-TβRI-ligand complex forms, the kinase within TβRII phosphorylates a juxtamembrane region (designated the GS box) of TβRI leading to the kinase activation of TβRI. The immunophilin FKBP12 tightly associates the TβRI at the Leu-Pro sequence near the GS box [159,160], preventing the ligand-independent activation of TβRI by TβRII without affecting their physical association [161,162]. Ligand binding to TβRII and the subsequent interaction of TβRII with TβRI ejects FKBP12 from TβRI, thereby permitting TβRII to bind to, serine transphosphorylate and activate TβRI. Although TβRII transphosphorylates TβRI largely at serine residues, these receptors also undergo tyrosine phosphorylation, enabling the recruitment of the SH2 (Src-homology 2) adaptor Shc (SH2-containing A2 protein) to TβRI (Figure 3) [163,164].

Inactivating germline mutations in TβRI have been reported in cancers of the breast, pancreas, biliary tree, cervix, and head and neck as well as chronic lymphocytic leukemia [165,166], whereas somatic inactivating mutation in TβRII occurs in multiple cancers, which include colorectal, gastric, endometrial, prostate breast, lung, liver, pancreas, cervical cancer as well as glioma and lymphoma [31,33,167,168]. As expected, TβRII knockout mice phenocopy TGF-β-deficient embryos, with embryonic lethality and aberrant hematopoiesis (193). Consistent with observations that certain tumor suppressor genes are silenced by gene promoter methylation, the loss of TβRI or/and TβRII expression occurs by their promoter methylation in multiple cancers, including esophageal squamous cell carcinoma [169], gastric adenocarcinoma [170], head and neck squamous cell carcinoma [171,172], colorectal cancer (CRC) [173], multiple myeloma [174], human B-cell lymphoma [175], renal cancer [176], breast cancer [177], and cell lung cancers [178,179]. Other mechanisms operant in cancer cells suppress the expression of TGF-β receptors. For example, an over-activation of the androgen receptor in prostate cancer cells suppresses the transcriptional expression of TβRII by suppressing the interaction of SP1 with the TβRII promoter [180].

## 7. Smads and Transcriptional Control

Following activation, TβRI proceeds to phosphorylate two serine residues in the C-termini of Smads 2 and 3, promoting their activation, multimerization (mainly heterotrimers with Smad4), and nuclear migration along with that of Smad4. Smads 2 and 3 collaborate with other transcriptional regulators, most notably Smad4 while binding to specific promoter response elements known as Smad binding elements (SBEs). Smad4 plays a critical role in the transcriptional activation of many but not all TGF-β-induced, Smad-dependent gene changes [5].

The Smad family comprises a group of highly conserved proteins characterized by their structural features. These proteins consist of an N-terminal MH1 domain promoting DNA binding, a C-terminal MH2 domain facilitating protein interactions, and a less conserved linker region between MH1 and MH2 domains [181]. In mammals, eight distinct Smads have been identified. These can be functionally categorized into R-Smads (receptor-activated Snads) Smads 1, 2, 3, 5, and 8), 2) co-Smad (Smad4), and 3) inhibitory Smads (Smads 6 and 7) [182,183,184].

Differences can be noted in the MH1 domain of Smad2 compared to other R-Smads, primarily due to a 30 amino acid insertion in Smad2. This sequence hinders direct binding to SBEs [134,185]. The Smad family is further subdivided based on their involvement with specific ligand subfamilies. Smads 2, 3, 4, and 7 mediate/modulate signal transduction by TGF-βs as well as the activin, and inhibin subfamilies, while Smads 1, 5, 8, and 6 mediate/modulate signal transduction by the BMP subfamily. Smad4, on the other hand, serves all subfamilies. It is worth noting that while a high degree of specificity exists in the activation of R-Smads by different receptors, exceptions exist.

Specific interactions between R-Smads and various TβRIs (ALKs 5, 4, and 7) are dictated by structural elements like the L45 loop of TβRI and the L3 loop of Smads 2 and 3 [186,187]. Smads 2 and 3 are recruited to TβRI through accessory proteins such as a Smad anchor for receptor activation (SARA), and hepatocyte growth factor-regulated tyrosine kinase substrate (Hgs/Hrs) (Figure 3) [188,189]. Subsequently, the TβRI phosphorylates two C-terminal serines at SSXS domains of R-Smads, facilitating their homo or heterodimerization. This dimerization enables their nuclear transport with Importin-β, which occurs once conserved nuclear localization signal (NLS) motifs are exposed [190,191]. Apart from SARA and Hrs, other proteins that bind to R-Smad, such as DAB2 (Disabled-2) and cPML (cytoplasmic promyelocytic leukemia protein), play crucial roles in facilitating the activation of R-Smads by promoting their delivery to TGF-β receptors (Figure 3) [192,193]. Normally localized in the nucleus, cPML is sequestered by various complexes, including the transcription factor c-Jun and the transcriptional repressor TGIF (TG-interacting factor). Upon stimulation by TGF-β, PCTA (a PML competitor for TGIF association) is translocated into the nucleus, where it competes with cPML for binding to TGIF. This competition enables the export of cPML to the cytoplasm, where it binds to R-Smads, thereby enhancing R-Smad-TβRI interaction [193]. 

Once in the cell nucleus, Smads 3 and 4 take on the task of controlling the transcription of a variety of genes targeted by TGF-β, which in turn mediate various cellular functions [194,195,196]. Notably, Smads exhibit a low affinity for SBEs. Consequently, their binding to promoters and enhancers is predominantly dependent on interactions with adjacent response elements close to SBEs, facilitated by other DNA-binding proteins [197]. To control gene expression, Smads cooperate with a diverse array of proteins, including transcription factors and co-regulators. This group encompasses AP-1, p300/CBP, HDAC, P/CAF, TGIF, Ski, Sno, MSG1, SNIP, and steroid hormone receptors [52,194,198]. Multiple kinases, such as ERK, JNK, p38-MAPK, CDKs 2, 4, 8, and 9 play a role in regulating TGF-β signaling by selectively phosphorylating the linker region of R-Smads [199]. Some of these kinases also phosphorylate the cytoplasmic domain of TGF-β receptors promoting alternative signaling pathways beyond the Smads (non-canonical pathways) [5,200]. The phosphorylation of R-Smads by CDKs 8 and 9 generates docking sites for YAP (Yes-associated protein) and PIN1 (peptidylprolyl cis/trans isomerase, NIMA-interacting 1) which enhances the transcriptional activity of R-Smads [5].

While both Smad2 and Smad3 are activated by TβRI, and most changes in gene expression depend on Smad3 more so than Smad2, germline knockout mice studies illustrate that each Smad has a unique role in mediating TGF-β responses. Although the germline knockout of Smad2 exhibits early embryonic lethality [201,202,203], Smad3 knockout mice are viable, have reduced body mass, are immune deficient, and develop metastatic CRCs [204,205,206,207]. Smad4 germline knockout mice exhibit early embryonic lethality [208,209]. Inactivating mutations in Smads 2 and 3 have been reported in a variety of cancers, although their incidence is lower than mutations in the TGF-β receptors [210]. In prostate cancer, the reactivation of the androgen receptor represses the Smad3 gene promoter activity [211]. Evidence also supports that the hyperactivation of Smad3 is associated with poor cancer prognosis. One mechanism for such activation in cancers is through the histone methyltransferase EZH2 (enhancer of zeste homolog 2), a novel cancer therapeutic target. EZH2, which is activated in cancers, promotes the methylation of Smad3, facilitating the recruitment of Smad3 to SARA and Smad3′s subsequent activation by TβRI [212]. Given their critical role in TGF-β signaling, Smads are potential therapeutic targets in cancer and fibrosis.

TGF-β signaling is under tight negative feedback control. It is rapidly deactivated through the concerted actions of inhibitory Smad7, the targeted degradation of TGF-β receptors and Smads via ubiquitin ligases like HECT, Smurfs, ROC-1, and Arkadia [213,214,215,216], as well as the deactivation of R-Smads mediated by the nuclear phosphatase PPM1A (Figure 3) [217]. Readers are referred to a recent review by Runa et al. [218] for a more in-depth look at the post-translation control and nuclear uptake of Smads in cancer. 

## 8. Non-Canonical Pathways of TGF-β Signaling

TGF-β signaling has been described to occur through both canonical and non-canonical pathways, each presumed to be distinct except for signaling through the same sets of TGF-β receptors. While the canonical pathway is mediated through Smads, the non-canonical pathway is independent of Smads [11,164,219,220] and mainly involves the MAPK and PI3K pathways, controlling the activation of ERK, JNK, p38-MAPK, and AKT (Figure 3) [164,220,221]. Although the canonical TGF-β signaling pathway plays a crucial role in suppressing tumorigenesis through various mechanisms including the suppression of cell proliferation and apoptosis [8,11,222,223], the non-canonical pathway is largely pro-tumorigenic. The molecular mechanisms by which the non-canonical kinases are activated, although incompletely understood, are not mediated by TGF-β receptor-Smad interaction. The membrane anchor of PI3K by TGF-β receptors leads to the activation of AKT followed by mTORC1, while the binding of TGF-β receptors to the adaptor protein TRAF6 couples TGF-β receptors to TGF-β activated kinase 1 (TAK1), p38-MAPK, and JNK (Figure 3) [221,224], which control cell proliferation and cell survival. The tyrosine phosphorylation of TβRI by TβRII upon TGF-β binding serves as a docking site for the recruitment of ShcA, which leads to the recruitment and activation of the GEF (GTP exchange factor) SOS (son of sevenless), promoting the activation of Ras and downstream signaling cascade, namely Raf, MEK, and ERK1/2 [163,164]. Additionally, TβRII directly phosphorylates the tight junction regulator PAR6, leading to changes and increased cell migration following the breakdown of tight junctions [225,226]. Other non-canonical routes are associated with cell survival, motility, and cytoskeletal reorganization. These include Rho-like GTPases and c-Src, affecting the actin cytoskeleton and cell migration [219]. 

While the non-canonical TGF-β signaling pathways may not drive transcriptional targets of Smads, Smads may influence components of these pathways through direct physical interactions. Moreover, many of the kinases activated by non-canonical TGF-β signaling modulate Smad function [199,227,228]. However, the collaboration between canonical TGF-β signaling and its non-canonical counterparts in late-stage cancer remains unclear. Both pathways are essential for TGF-β-induced epithelial-mesenchymal transformation (EMT) in mammary epithelial cells (MECs), and crosstalk between Smad2/3 and non-canonical effectors like Ras drive EMT and metastasis. Evidence supports that sustained EMT, triggered by TGF-β, reduces Smad3 expression through various non-canonical effectors. The molecular outcomes of Smad2/3 signaling are controlled by phosphorylation, with activated Ras impacting nuclear translocation. Additionally, various protein kinases influence Smad2/3 function in response to TGF-β. These complexities underscore the importance of understanding the molecular connections between Smad2/3 and non-canonical equivalents in normal and malignant cells to decipher TGF-β’s role in both normal biology and pathology. 

## 9. Normal Functions of TGF-βs

TGF-βs exert pivotal roles in overseeing a wide array of normal cellular, physiological, and developmental functions; they exert functional versatility across various cell types, tissues, and organ systems, with their effects intricately contingent on context [229,230]. These encompass the regulation of cell proliferation, apoptosis, survival, differentiation, senescence, autophagy, extracellular matrix production, wound healing, cell adhesion, cell migration, epithelial-mesenchymal transition (EMT), chemotaxis, immune regulation, invasion, muscle and bone development, mesoderm induction, angiogenesis, and immune modulation [2,231,232,233,234]. Here we will provide the mechanistic basis for some of the key functions of TGF-β pertinent to cancer and fibrosis.

### 9.1. Suppression of Proliferation

Regarding their proliferative impact, TGF-βs typically prompt growth arrest in normal epithelial cells, while conversely fostering the survival/expansion of neurons and stromal fibroblasts. TGF-βs’ growth arrest mechanisms are largely reliant on Smad-dependent processes. These involve the downregulation of diverse cyclins and cyclin-dependent kinases, coupled with the upregulation of cyclin-dependent kinase inhibitors [195,235]. TGF-β1 also suppresses growth by the downregulation of cdc25A by involving HDAC recruitment through E2F-p130 interactions [236]. Moreover, TGF-β1’s ability to suppress growth emerges from its downregulation of the c-Myc proto-oncogene, thereby liberating its interaction with Miz-1 and Max. Miz-1 then transcriptionally activates p15INK4b, which inhibits the CDK4-cyclin D complex [237,238].

### 9.2. Induction of Apoptosis

TGF-βs induce apoptosis in diverse cell types, often through a spectrum of related mechanisms. Some mechanisms require both Smads and AP-1 [196]. The apoptotic response initiated by TGF-β involves the activity of various caspases, encompassing both the intrinsic and extrinsic apoptotic pathways [239,240]. The apoptotic mechanisms of TGF-β encompass the upregulation of pro-apoptotic members within the BCL2 family, along with the downregulation of their anti-apoptotic counterparts [240,241]. This cascade triggers the release of cytochrome c from mitochondria, thereby activating caspases 9 and 3 [241]. Additional mediators/modulators of TGF-β that induce cell death include DAP kinase [242], TAK-1 [243], Daxx [244], NF-kB [245], Smad7 [246], Bim [247], GADD45b [248], survivin, Bcl-xl, and FLIP [240,249,250,251]. Generally, the precise mechanisms governing TGF-β-induced growth arrest and apoptosis are both cell-type and tissue-dependent.

### 9.3. Role of TGF-β1 in the Immune System

**Figure 4:**
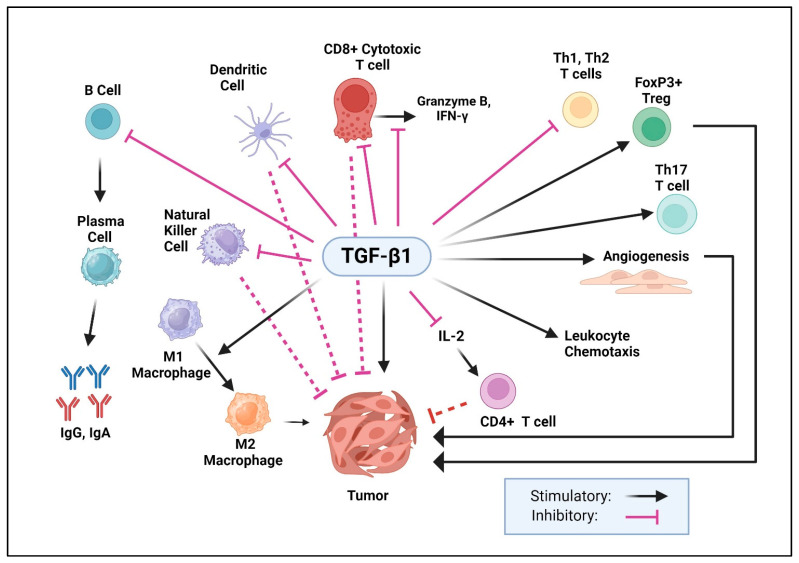
**Role of TGF-β1 in immune regulation.** The intricate role of TGF-β1 in immune regulation is depicted, showcasing its dual nature as both an inducer of immune tolerance and a regulator of immune effector functions. TGF-β1 transforms CD4+ T cells into regulatory T cells (Tregs), essential for immune homeostasis. TGF-β1 also exerts inhibitory effects on CD8+ cytotoxic T cells, natural killer cells, and antigen-presenting cells, dampening their effector functions. Additionally, it suppresses B cell differentiation and antibody production, further contributing to immune regulation. On the other hand, TGF-β released by tumor cells promotes angiogenesis, promotes leukocyte chemotaxis, and promotes the differentiation of macrophages from an M1 to an M2 phenotype. Both M2 macrophages and angiogenesis promote tumor growth.

TGF-β1 plays a pivotal role in immune response regulation (Figure 4), which became first evident through initial investigations in TGF-β1 germline null mice. These mice exhibited early postnatal mortality with multiorgan inflammation resembling an autoimmune disorder [57]. Subsequent studies demonstrated the rescue of this phenotype through deficiency in either major histocompatibility complex (MHC) class II [252] or β2-microglobulin [253]. These and other studies collectively suggested that the absence of TGF-β1 results in an uncontrolled adaptive T-cell response. An autoimmune phenotype also occurred in mice with T cell-specific deletions of TβRII [254], TβRI [255], or TGF-β1 [256]. These manifestations were attributed to CD4+ T cell activation by self-antigens [257]. Altogether, these groundbreaking studies established the critical role of TGF-β1 in the acquisition of T cell tolerance during thymic development. 

Subsequent studies showed that TGF-β1 induces the differentiation of CD4+ T cells into regulatory T cells (Tregs), which are instrumental in maintaining immune homeostasis [258]. Upon TGF-β1 activation, Smads synergize with STAT5 (signal transducer and activator of transcription 5) and NFAT (nuclear factor of activated T cells) to induce FOXP3 (forkhead box P3) expression in naive CD4+ T cells, stimulating their differentiation into Treg cells [259]. Additionally, in collaboration with RORγ2 (RAR-related orphan receptor γ2), Smads induce a T helper 17 (TH17) phenotype [260,261]. TGF-β1 also hampers the function of CD8+ cytotoxic T cells, NK (natural killer) cells, and antigen-presenting cells, such as dendritic cells and macrophages [3,262]. Upon TGF-β1 stimulation of CD8+ T cells, Smads work in partnership with the transcription factor ATF1 (activating transcription factor 1) to suppress the expression of several cytolytic genes such as granzyme B and IFN-γ (interferon γ) [263]. Additionally, TGF-β1 suppresses the expression of IL-2, a cytokine promoting the proliferation of CD4+T cells [264]. Moreover, TGF-β1 inhibits B cell differentiation and function [2,265,266], thereby limiting antibody production. In conjunction with the transcription factor RUNX3 (runt-related transcription factor 3), Smads play a regulatory role in immunoglobulin class switching in B cells [267]. 

### 9.4. Role of TGF-β1 in Wound Healing

**Figure 5:**
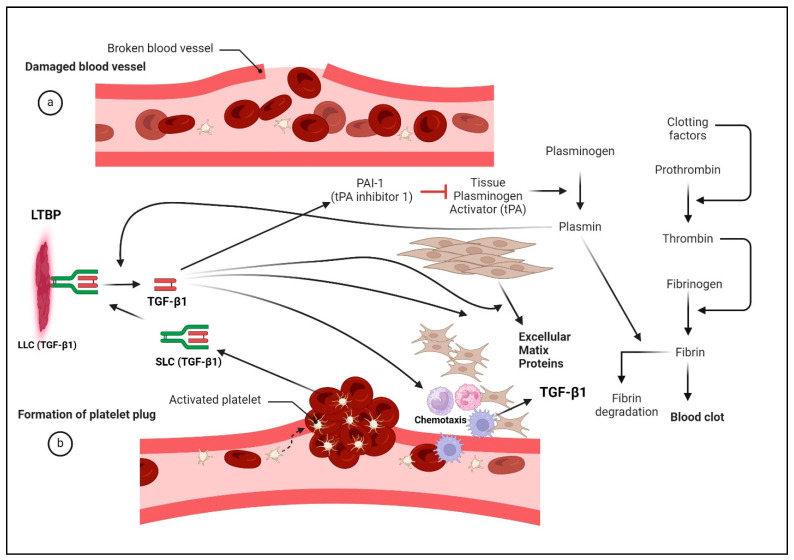
The intricate involvement of TGF-β in wound healing, shedding light on its diverse roles and the complex interplay with various cellular processes. Platelets emerge as pivotal players, releasing TGF-β1 upon degranulation at the wound site, where it orchestrates a cascade of events. Tissue plasminogen activator (tPA) cleaves plasminogen into plasmin, which not only acts to limit the size of a blood clot by breaking down fibrin but also functions to activate TGF-β1 by cleaving it from its large latency complex (LLC). Here, activated TGF-β1 then acts as a chemoattractant for immune cells while simultaneously stimulating fibroblast proliferation and differentiation into myofibroblasts, which contribute to extracellular matrix deposition and wound repair. Despite its role in promoting wound repair, TGF-β1 induces immunotolerance, crucial for dampening autoimmunity triggered by tissue damage in normal tissue repair. TGF-β1 also drives the transcriptional induction of PAI-1 (tPA inhibitor-1), which functions to block the activation of plasmin, thereby limiting the extent of fibrin degradation. Tumors in which TGF-β1 is overexpressed/overactivated likely result in excess PAI-1 induction, which inhibits fibrin dissolution, thereby contributing to increased hypoxia and tissue damage.

The multifaceted and seemingly conflicting roles of TGF-β1 in cancer and fibrosis can perhaps find clarity through similarities to the processes of tissue injury and wound repair (Figure 5). Notably, platelets are endowed with a substantial source of TGF-β1, promptly releasing and activating it at the wound site following platelet degranulation [21,268]. Human and bovine platelets are essentially devoid of TGF-βs 2 or 3 [46,50]. TGF-β1 released upon platelet degranulation functions as a chemoattractant, luring monocytes, macrophages, and fibroblasts [269], while concurrently spurring fibroblasts to proliferate and differentiate into myofibroblasts [270]. These specialized cells express diverse extracellular matrix proteins like fibronectin and type I collagen [270]. This signal is subsequently amplified through the autoinduction of TGF-βs. Despite its role in chemotaxis to combat microbial infections, TGF-β1 simultaneously wields potent immunosuppressive effects, curbing autoimmunity triggered by tissue damage (114). Mice deficient in TGF-β1 exhibit delays and deficiencies in wound healing [271]. Counterintuitively, Smad3 knockout mice display enhanced wound healing, attributed to heightened re-epithelialization rates and significantly reduced local inflammation [207]. Such loss of the local inflammation is due to a suppression of TGF-β1-induced chemotaxis. This suggests that wound reparative processes, including chemotaxis and ECM deposition during wound healing, are suppressed by Smad3 [207]. In contrast, fibrosis induced by multiple agents is Smad3 dependent [272,273,274,275,276,277]. Collectively, these studies support that Smad3-induced chemotaxis suppresses wound repair but not fibrosis, which is dependent on or driven by Smad3. 

## 10. Mechanism of TGF-βs-Induced Tumor Progression

The mounting body of evidence supports that while TGF-βs serve as a tumor suppressor in normal epithelial cells, in late-stage cancers, where the tumor suppressor function of TGF-βs is subdued or eliminated, TGF-βs’ oncogenic functions not only get turned on but also dominate, thereby driving tumor growth, progression, and invasiveness [8]. Similar to the mechanisms controlling TGF-βs’ tumor suppressor activity, the mechanisms underlying the pro-tumorigenic functions of TGF-βs involve multiple discreet and interacting components. The oncogenic activity of TGF-βs occurs through both intrinsic (directly on the tumor cells) and extrinsic (indirectly on the tumor cells but mediated by tumor-associated cells or host response) mechanisms. 

### 10.1. Intrinsic Mechanisms of Tumor Promotion

The intrinsic mechanisms for the oncogenic function of TGF-βs necessitate that tumor cells have functional TGF-β receptors, particularly TβRI and TβRII, despite reduced numbers of receptors per cell or the complete loss of those receptors from certain tumor cell lineages. The increased availability of TGF-β ligands in tumors, particularly TGF-β1 (expressed by tumor cells, stromal elements, or released by platelets) coupled to the reduced sequestration of TGF-βs by EMC proteins such as decorin, and the enhanced activation of TGF-βs by factors such as proteases, integrins, and TSP-1, drives TGF-β signaling in tumor cells despite the loss of TGF-β receptor numbers. Additionally, late-stage cancer cells display altered TGF-β signaling, dampening or negating the growth-inhibitory or apoptosis-inducing effects of TGF-β, which in turn favors the balance towards oncogenic TGF-β signaling. A loss of TGF-β-induced growth arrest and apoptosis may occur either from the loss of downstream targets of growth arrest or apoptosis (common occurrences in cancers) or from disruption in TGF-β signaling mediators that control their expression or function. 

**EMT.** The intrinsic mechanisms behind the pro-tumorigenic function of TGF-βs involve the altered expression of genes associated with EMT, which promotes cell motility, invasiveness, and metastasis. TGF-β1 induces EMT through a Smad2/3-dependent mechanism involving the transcriptional induction of transcription factors Snail, Slug, and Twist, which in turn suppress the expression of epithelial markers like E-cadherin and stimulate the expression of mesenchymal markers like N-cadherin and vimentin [8,278,279]. This orchestrated molecular program leads to a phenotypic shift in cells, resulting in the loss of epithelial characteristics, increased cell motility, and enhanced invasiveness. TGF-β1 also induces the expression of integrin αvβ3, which conveys mammary epithelial cells with increased migratory/invasive phenotype by binding to TβRII and promoting the phosphorylation of TβRII at Y284 by c-Src, leading to the activation of p38-MAPK [280,281,282]. The overall impact is the transformation of epithelial cells into mesenchymal-like cells, a process crucial for embryonic development, and wound healing, but unfortunately, often exploited in pathological conditions such as cancer when dysregulated. The pro-tumorigenic function of TGF-β1 also occurs directly on tumor cells despite having reduced levels of TGF-β receptor and Smads. As the intrinsic tumor suppressive pathways of TGF-βs are largely dependent on Smads, a loss of Smads in cancer cells favors non-canonical TGF-β pathways that drive invasion, metastasis, aggressiveness, and therapeutic resistance [12].

**Cancer Stem Cells (CSCs).** CSCs are an “elusive” subset of cells with self-renewal abilities, contributing to cancer initiation, recurrence, and heterogeneity of both primary tumors and distant metastases [283,284]. Importantly, CSCs are often quiescent/dormant or slow-growing cells that are resistant to traditional chemotherapies, which instead target rapidly dividing cells. This resistance is believed to be responsible for treatment failure or relapse and tumor growth from minimal residual disease [285]. Efforts to improve cancer therapy involve developing strategies to target and eliminate both CSCs and non-CSCs, aiming to enhance treatment effectiveness and prevent tumor recurrence. It is well-recognized that EMT can induce CSC-like cells with both stem cell and non-stem cell characteristics [286,287] and that EMT and CSC-like traits promote metastasis and resistance to chemotherapeutic drugs [288,289]. Accumulating evidence underscores the connection between TGF-β signaling and the development and persistence of CSCs in carcinomas. For example, CD44+/CD24− breast carcinoma cells with CSC-like properties showed an enhanced TGF-β signaling signature compared to their non-CSC-like (CD44−/CD24+) counterparts [283]. Moreover, the inhibition of the TβRI kinase suppresses this CSC-like pool, emphasizing TGF-β’s role in stem-cell maintenance [283]. Supporting this notion, reduced CSC markers were observed in tumor cells from a patient with glioblastoma (GBM) in a clinical trial with a TβRI kinase inhibitor [290]. TGF-β-activated Smad2 has been implicated in sustaining this CSC phenotype through EMT [291]. In hematological malignancies, TGF-β signaling suggests leukemia-initiating cell maintenance in CML [292]. BCR-ABL-positive CML patients’ hemangioblasts overexpress TGF-β1, creating an immune-protected milieu for stem cells [293]. Consequently, TGF-β inhibitors may be uniquely effective in targeting and disrupting EMT and CSCs, making them particularly appealing to oncologists.

**TGF-β Oncogenic Switches.** Significant evidence suggests that a specific molecular relay or a discrete set of switches exists during the process of carcinogenesis that toggles TGF-β’s function from tumor suppressor to tumor promoter. Some of these switches involve the loss or suppression of R-Smad signaling, which favors non-canonical/tumor-promoting TGF-β signaling. For example, the epigenetic downregulation of DAB2 by promoter methylation in squamous cell carcinomas (SCCs) acts as a TGF-β oncogenic switch [294]. DAB2 downregulation, which results in suppressing R-Smad/canonical TGF-β signaling, not only correlates with poor prognosis but also fundamentally alters the TGF-β response from a tumor suppressor into a potent promoter of migration, anchorage-independent growth, and in vivo tumor growth. Similarly, the focal adhesion adaptor p130Cas (Crk-associated substrate, 130 kDa), which is overexpressed in breast cancer cells, promotes TGF-β1-induced EMT by binding to Smad3/TβRI, promoting the degradation of Smad3 and thereby suppressing the cytostatic activities of Smad3 [295,296]. Various other non-canonical TGF-β1 signal transducers, such as GSK-3β and NF-κB, also suppress the expression of Smad3 (canonical TGF-β1 signaling) in breast cancer [297,298,299]. Oncogenic Ras, which promotes TGF-β1-induced EMT, activates ERK1/2, which phosphorylates the middle-linker of Smad3, suppressing Smad3′s nuclear translocation and cytostatic function [300]. Non-canonical TGF-β1 signaling also activates ERK1/2 (Figure 3), similarly suppressing Smad3′s transcriptional responses. 

Another intriguing facet of the TGF-β1 switch is evident in breast cancer, where the TGF-β1-induced transmembrane prostate androgen-induced protein (TMEPAI), which is highly expressed in various types of cancers, has been proposed to act as a TGF-β oncogenic switch [301]. TMEPAI, induced by TGF-β1, provides negative feedback on TGF-β1 signaling by interacting with Smad2 and preventing SARA from recruiting Smad2 to TβRI [302], and hence, suppresses the conical tumor suppressor action of TGF-β1. The TGF-β1-induced TMEPA1 also promotes the growth and invasiveness of cancer cells partly through downregulating the expression of the tumor suppressor PTEN, leading to the activation of PI3K, and inhibiting the expression of PHLPP1 (PH domain and leucine-rich repeat protein phosphatase 1) with the subsequent activation of AKT [303]. In yet another study on breast cancer, the tumor suppressor function of the ubiquitin ligase PHRF1 was identified to occur through a ubiquitin-mediated decay of the homeodomain protein TGIF [304], a suppressor of the canonical TGF-β signaling pathway that directly represses cPML’s ability to enable SARA-dependent transport of Smad2 to TβRI (Figure 3). The loss or silencing of PHRF1 in breast cancer disrupts the TGF-β/Smad cytostatic program. 

Further insight into the TGF-β oncogenic switch in pancreatic ductal adenocarcinoma (PDA) was developed by an intriguing study published in Cell [9]. This study showed that the loss of Smad4, a frequent event linked to the progression of PDA, disables TGF-β1-sensitive PDA cells from their normal fate of undergoing a lethal program of EMT towards promoting tumor growth. According to this model, the loss of Smad4 converts Sox4 from an inducer of apoptosis to a TGF-β1 tumor promoter. This switch is facilitated by an EMT-linked remodeling of the transcription factor landscape, including the de-repression of Klf5. This study underscores that the oncogenic switch of TGF-β1 in PDA operates through an EMT-mediated disruption of a lineage-specific transcriptional network. Through another mechanism, in pancreatic ductal cells, zinc-alpha2-glycoprotein (AZGP1) functions as a tumor suppressor by inhibiting TGF-β-mediated EMT [305]. The expression of AZGP1 is epigenetically repressed in PDA by histone deacetylation, thereby enabling TGF-β-induced EMT, apparently through the non-canonical TGF-β activation of ERK [305]. AZGP1 is also lost in other cancers, enabling TGF-β1-induced EMT [306,307]. Lenvatinib, an FDA-approved anti-cancer small chemical inhibitor of multiple tyrosine kinases, induced AZGP1 expression in cholangiocarcinoma where it led to the suppression of TGF-β1-induced EMT [306]. Consistent with its role in modulating TGF-β signaling, the overexpression of AZGP1 was shown to prevent fibrosis in a mouse model of kidney fibrosis [308].

In CRC, the TGF-β-responsive gene NDRG2 (N-Myc-downstream-regulated gene-2) appears as a critical factor that counteracts TGF-β1-induced EMT. NDRG2 level in normal colonic cells is elevated by TGF-β1 through disrupting the binding of the repressive c-Myc/Miz-1 complex on the NDRG2 promoter. The levels of NDRG2 are lost in CRC, thereby enabling TGF-β-induced EMT [309]. NDRG2 also functions as a tumor suppressor whose expression is lost in other cancers [310], in which its enforced overexpression suppresses the expression of EMT markers [311]. The knockdown of NDRG2 also promotes TGF-β1-induced fibrotic markers in renal tubular epithelial cells [312].

Another study reported that the loss of the pioneering transcription factor FOXA1 (forehead box A1) in nasopharyngeal carcinoma reprograms a genome-wide network of TGF-β1-regulated genes from driving tumor suppression to driving EMT and cell proliferation [313]. In line with this, FOXA1 induces the expression of the TGF-β-responsive tumor suppressor BAMBI by promoting the binding of Smad2/3 to the BAMBI promoter [313]. An overexpression of BAMBI alone suppresses the proliferation, migration, and invasiveness of nasopharyngeal carcinoma cells. This showcases the complexity of TGF-β1’s actions, with FOXA1 playing a crucial role in restoring sensitivity to TGF-β1’s growth-inhibitory effects. 

TrkB, a receptor tyrosine kinase for brain-derived neurotrophic factor (BDNF), also disrupts TGF-β1’s tumor suppressor activity in various cancers [314]. The activation of TrkB inhibits TGF-β1-mediated tumor suppression by interacting with R-Smad/Smad through complex interactions with downstream effectors. Overall, these findings collectively contribute to a nuanced understanding of the intricate molecular switches that govern TGF-β’s dual roles in cancer and further provide new therapeutic targets.

### 10.2. Extrinsic Mechanisms of Tumor Promotion

In cases of genetic aberrations in cancers, such as truncating mutations affecting the initiation of TGF-β receptor signaling, like the TβRII frame-shift mutation [31], the TGF-β signaling pathway can be disrupted, thereby tipping the balance towards the extrinsic pro-tumorigenic functions of TGF-βs. These appear to be mediated by multiple mechanisms within the TME, mainly those that exploit TGF-βs’ action on compromising the tumor immune surveillance system [3]. Numerous key findings underscore the immunosuppressive role of TGF-β1 in tumor progression. Transgenic mice expressing dominant-negative TβRII in CD4+ and CD8+ T cells exhibit resistance to tumor growth, indicating the necessity of TGF-β in those T cell lineages for tumor growth [315]. This resistance is linked with a marked increase in tumor-reactive CD8+ T cells [315]. TGF-β1 secreted by T cells drives tumor evasion from adaptive immunity in a prostate cancer model [316]. TGF-β signaling inhibits the priming of tumor-antigen-specific T cells and attenuates the effector function of CD8+ cells in melanoma patients [317]. Additionally, TGF-β induces a T cell regulatory phenotype, promoting the differentiation of immunosuppressive Tregs [318]. Correlations between TGF-β levels and FoxP3 expression in transcriptomic datasets suggest its involvement in Treg induction in skin cutaneous melanoma and breast cancer [319]. Moreover, the TGF-β-induced inhibition of dendritic cell antigen presentation and suppression of NK cell function further contribute to immune evasion [320]. Macrophages in the TME polarize towards an M2 phenotype under the influence of TGF-β, further promoting an immunosuppressive milieu [321,322]. The net result of these multifaceted immunosuppressive effects of TGF-β dampens host immune surveillance against cancer cells.

Additional extrinsic oncogenic mechanisms of TGF-βs involve carcinoma-associated fibroblasts (CAF) [323,324] and vascular endothelial cells [325,326]. TGF-β1 secreted by tumors promotes the transdifferentiation of adjacent fibroblasts into myofibroblasts with properties that support the growth, survival, and progression of cancer [327]. While TGF-β receptors are either lost or downregulated in most if not all cancers, TGF-β ligands, particularly TGF-β1, are upregulated in carcinomas, as detailed earlier. This induced expression of TGF-β1 in cancer cells, along with the enhanced expression of enzymes (plasmin, MMPs) and other cancer-associated triggers that activate latent TGF-β1 (integrins and TSP-1, ROS), as described earlier, serve tumors with elevated levels of active TGF-β1 that aid cancer cells to grow, survive, and metastasize. Moreover, as will be covered later in detail, carcinomas promote a hypercoagulable state in the host, leading to enhanced thrombosis [328], which results in the release and activation of platelet TGF-β1 as well as other platelet growth factors. The importance of this cannot be overstated, given that patients with cancer have a 9-fold increased risk of venous thromboembolism, which is also the second leading cause of cancer deaths [328]. 

## 11. Role of TGF-βs in the Pathophysiology of Fibrosis

**Figure 6:**
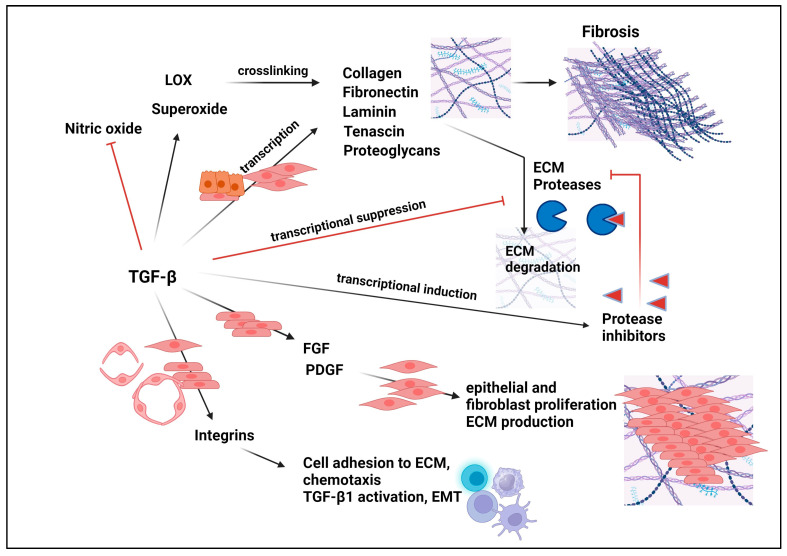
**The central role of TGF-βs in driving fibrosis.** TGF-βs induce extracellular matrix (ECM) production by driving the transcription of genes for the expression of ECM proteins such as collagen, fibronectin, laminin, tenascin, and proteoglycans, while it also inhibits ECM breakdown by inhibiting the transcription of ECM proteases and inducing the expression of ECM protease inhibitors. Elevated TGF-β levels in certain pathologies including cancers contribute to tissue fibrosis, by overproduction and over-activation of TGF-β and TGF-β signaling. TGF-β also induces the expression of lysyl oxidase (LOX) genes, which promotes the crosslinking between EMC proteins, contributing to ECM rigidity. LOX and superoxide (ROS) promote TGFβ-induced fibrosis. In desmoplastic cancers, excess ECM promotes metastasis and activates latent TGF-βs, further exacerbating fibrosis and impeding the efficacy of chemotherapeutic drug access and drug resistance.

Fibrosis is a common pathological manifestation of chronic inflammatory conditions, which include malignancies [329,330,331]. In these conditions, normal homeostatic mechanisms are seriously impaired and chronic inflammation triggers a cascade of events involving immune cells, fibroblasts, and epithelial cells. Those conditions promote the production of pro-inflammatory cytokines and profibrotic growth factors such as TGF-β, PDGF, and tumor necrosis factor-α (TNF-α), which promote the accumulation and activation of fibroblast, promoting excessive extracellular matrix (ECM) production, which, over time, results in tissue remodeling, scarring, and fibrosis [332]. TGF-β1 is a prominent profibrotic cytokine well-documented to induce the synthesis and deposition of ECM proteins including fibronectin, tenascin, collagens, and proteoglycans (Figure 6) [333,334]. The induced expression occurs either through either Smad-dependent or Smad-independent (non-canonical) TGF-β signaling [335,336,337]. TGF-β1 also hinders ECM breakdown by reducing protease synthesis and elevating levels of protease inhibitors (Figure 6) [334,338]. While an elevated deposition of ECM is a critical part of TGF-β1′s action in normal wound healing, the overexpression and over-activation of TGF-β1 in conjunction with other profibrotic cytokines collaborate to drive tissue fibrosis. Shear stress from hypertension can induce TGF-β1 levels [74] and activate latent TGF-β1 [134]. Moreover, the over-activation of the renin-angiogenin-aldosterone system (RAAS), a major driver of hypertension, activates fibrosis by driving the expression of TGF-β1 [339]. This makes the TGF-β signaling pathway an attractive therapeutic target of fibrosis in multiple pathologies. 

The fibrotic response triggered by TGF-βs also plays a fundamental role in the progression of cancer. This is particularly evident in desmoplastic cancers characterized by excess ECM [7,340,341]. Desmoplasia not only promotes metastasis [342,343] but also activates latent TGF-βs 1 and 3 by tension exerted between integrins and ECM, leading to signal amplification and increased fibrosis [343]. TGF-β1 can induce the expression of lysine oxidases (LOXs), which cross-links collagens to elastin in ECM, leading to tissue tension/rigidity and promoting increased migration, EMT, and fibrosis [343,344,345,346,347,348,349]. Fibrosis not only impairs normal tissue architecture and function but blocks access to chemotherapeutic drugs in the inflicted pathological tissues and tumors [340].

**Figure 7:**
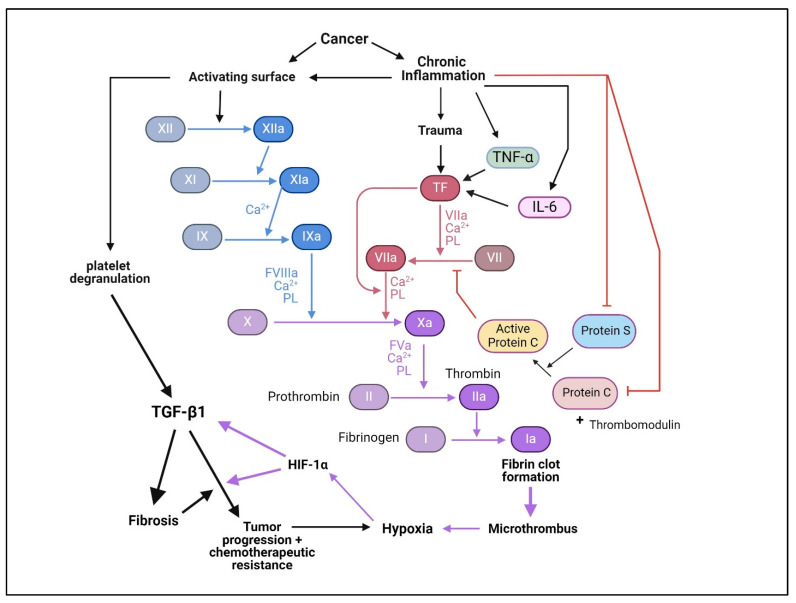
The intricate relationship between chronic inflammation, coagulation, and TGF-β1, particularly in the context of malignancies. Chronic inflammation can trigger the coagulation cascade through the activation of factors like tissue factor and Factor XII. This leads to the formation of microthrombi within blood vessels, causing hypoxia and tissue damage, further exacerbating inflammation and clotting. Platelets play a pivotal role in this process by releasing various substances, including pro-fibrotic factors like TGF-β1. Additionally, hypoxia-inducible factors (HIFs) driven by tumor hypoxia promote the stabilization of HIF-1α, which cooperates with TGF-β1 to drive fibrosis and tumor progression.

Another mechanism by which chronic inflammation promotes fibrosis involves the clotting pathway. The normal interplay between inflammation and coagulation is a crucial aspect of the body’s response to injury or infection, but when inflammation is chronic a complex series of events can trigger the onset of thrombosis (Figure 7) [350]. Chronic inflammation can enhance the coagulation cascade through the increased expression of pro-inflammatory factors (IL-6, TNF-α) [351,352], which in turn can trigger the expression of tissue factor (TF), a key initiator of the coagulation cascade. Inflammation can lead to the activation of coagulation factors that are usually in an inactive state in the blood. Moreover, inflammatory mediators can activate Factor XII (Hageman factor), which is part of the intrinsic pathway of coagulation [353].

Once activated, Factor XII initiates a series of reactions that ultimately lead to the activation of Factor X and the production of thrombin, a central player in clot formation [353]. Moreover, chronic inflammation can disrupt the balance between pro-coagulant and anticoagulant pathways. For instance, inflammation can suppress the production of natural anticoagulants like protein C and protein S, tipping the scale toward clot formation [354]. Prolonged inflammation can lead to the formation of microthrombi within blood vessels. These microthrombi can impede blood flow, causing hypoxia and tissue damage, potentially triggering a positive feedback loop where the resulting tissue damage leads to further inflammation and clotting. The resulting tissue hypoxia can then induce the expression of TGF-β1 [355] and activate TGF-β signaling to promote collagen expression by dermal fibroblasts (Figure 7) [356].

Chronic inflammation has been identified as a significant factor in the impairment of the endothelial lining within blood vessels [357]. This impairment contributes to the exposure of subendothelial collagen, initiating a cascade of primary hemostasis resulting from the activation of platelets, which not only aggregate at the site of injury but also release a spectrum of substances that promote inflammation, coagulation, and fibrosis. Among these substances are pro-inflammatory and pro-coagulant factors, along with pro-fibrotic factors such as TGF-β1 and PDGF. The released platelet-derived factors, particularly TGF-β1, play a crucial role in driving fibrotic processes. Notably, platelets are the richest tissue source of TGF-β1. To illustrate, about 10 mg of TGF-β1 has been purified from 2.5 g of outdated human platelets, accounting for over 0.4 mg/g of tissue [22]. This substantial release of TGF-β1 becomes particularly impactful when considering the minimal amount required for maximal biological activity. In line with this, the concentration of TGF-β1 in human serum derived from platelets was measured to be about 1.3 nM or 315 ng/mL [46], which is >31.5-fold in excess of its maximal activity (10 ng/mL).

Another mechanism of increased thrombosis that is operant in various malignancies involves hypoxia-inducible factors (HIFs). The rapid growth of tumor cells leads to a tumor hypoxic niche [358,359]. This hypoxic niche, in conjunction with the inherent metabolic rewiring of cancer cells, elevates HIFs in tumor cells that cooperatively induce the transcription of VEGF and TGF-β1, both serving to promote angiogenesis through binding to and stimulating the growth of endothelial cells (Figure 7) [356,359,360,361,362]. However, this angiogenic signal is so strong that tumors often become over-vascularized, causing contorted vasculature and a subsequent increase in hypoxia from poor or stagnant circulation [363]. Stasis from stagnant blood flow promotes thrombosis [364] and hence the release and activation of platelet-derived TGF-β1. Ultimately this increased TGF-β1 further enables tumors to escape immune surveillance, induce fibrosis, and through additional mechanisms promote tumor growth and aggressiveness. Indeed, HIF-1α and TGF-β1 cooperate synergistically to induce fibrosis in multiple tissues and the tumor progression of many cancers [365]. 

Due to their hypercoagulable state, many cancer patients are given antithrombotic drugs [328]. A common anticoagulant given to cancer patients is low molecular weight heparin (LMWH), which has been reported to inhibit tumor metastasis [366]. Preclinical and clinical studies support that LMWHs can also significantly inhibit inflammation and fibrosis [367,368,369]. How LMWH inhibits inflammation, tumor metastasis, and fibrosis is incompletely understood. These protective effects likely occur partly through blocking the release of platelet growth factors such as TGF-β1. 

Readers are referred to recent reviews and reports on the therapeutic targeting of TGF-β in renal, pulmonary, liver, and cardiac fibrosis [4,370,371,372,373] for further information about the role of TGF-β in fibrosis.

## 12. Current Approaches in the Therapeutics for Targeting TGF-β in Cancer

It is crucial to acknowledge that the clinical implementation of drugs targeting TGF-β signaling followed a cautious approach. This deliberate pace aimed to prevent the interference with TGF-β’s role as a tumor suppressor, considering the potential hazards such as the emergence of unrelated neoplasms, heightened growth of primary tumors, and the activation of dormant metastatic tumor cells [374]. Additionally, apprehensions surfaced from research indicating severe vascular and inflammatory complications in mice with suppressed TGF-β1 expression, prompting concerns about possible life-threatening side effects in humans [57,375]. Clinical trials faced prolonged halts due to the discovery of aortic aneurysms and hemorrhagic lesions in animals from TGF-β blockade [101,374,376]. Thereafter, efforts were made to develop biomarkers and conduct modeling for a more precise assessment of therapeutic windows [374,377]. 

Many different types of TGF-β signaling inhibitors have been developed and continue to be in development, an increasing number of which target discreet components of TGF-β signaling in cancers for a more targeted approach (Table 1). They are currently under investigation for both preclinical and early clinical phases. These strategies can be categorized into five distinct groups: receptor kinase inhibitors, ligand traps, monoclonal antibodies, antisense oligonucleotides, and aptamers (both peptide- and nucleotide-based ones). While many have significant clinical promise, most have significant drawbacks such as toxicity and limited therapeutic benefit. For many of them, their clinical usefulness depends on several factors, and their relative benefits and limitations need to be considered on a case-by-case basis. While further research is needed to better understand the optimal strategies for their use in different diseases and stages of cancer, several principles can be currently applied to promote their optimal clinical use.

### 12.1. Important Considerations

When considering TGF-β inhibitor therapies for cancer patients, it is prudent to conduct a thorough evaluation of various factors to best tailor treatment strategies [374]. First, the magnitude and isoform of TGF-β production by the tumor or its microenvironment is a critical parameter, and measuring circulating or biopsy-derived TGF-β isoform levels can guide treatment decisions regarding the optimal choice of the isoform-selectivity of a TGF-β ligand anti-antagonist. When gauging TGF-β isoforms, emphasis should be placed on measuring protein levels of TGF-β rather than solely relying on mRNA levels, as TGF-β isoforms undergo translational control [378,379]. Second, the activation of a TGF-β responsive gene expression signature within the tumor cells and tumor stroma including tumor-infiltrating immune cells (TILs) could be used to predict the potential impact of TGF-β blockade on outcome, and the choice of other therapeutic modalities that may work synergistically with TGF-β blockage. Third, it would be prudent to stain tumor biopsies for total- and P-Smad2^S465/467^ as well as other markers to assess active TGF-β signaling within tumors and to help gauge the TGF-β-responsive cell types. Fourth, the genetic makeup of the tumor should be scrutinized, focusing on the expression and mutations in TβRII, TβRI, and Smads, as well as their epigenetic silencing as these alterations can influence the response to the site of TGF-β inhibition. Various tumor characteristics play a pivotal role in treatment planning, given that late-stage cancers typically present a loss of TGF-β tumor suppressor function. Last, it could be helpful to assess the extent of host cellular responses, such as markers of local or systemic levels of immune regulatory cells affected by TGF-β, desmoplasia as well as tumor hypoxia, as they could help devise an optimal therapeutic strategy. Integrating these considerations ensures a comprehensive approach to TGF-β inhibitor therapies, enhancing precision and therapeutic efficacy for cancer patients.

### 12.2. Standalone versus Combination TGF-β Signaling-Blockade Therapies

TGF-β inhibitors in cancer therapeutics have been used as both standalone as well as in combination with other treatments. In most applications to date, treatment with TGF-β inhibitors alone has had limited efficacy relative to co-treatment with other therapies. While most combination therapeutics that work were identified empirically, patients would benefit if an optimal drug combination could be predicted in advance of treatment. One can predict the potential therapeutic effectiveness of a particular TGF-β inhibitor in conjunction with a conventional therapeutic by surveying the impact of the conventional therapeutic alone on the activation of a TGF-β gene expression signature within the tumor. This is important as some cancer therapies induce the expression and/or the activation of various TGF-β ligands. For example, radiation has been shown to both induce the expression [380] and activation of latent TGF-β1 [124], which may contribute to fibrosis and therapy resistance. The antineoplastic drug bleomycin sulfate used for some malignancies, induces the expression of TGF-β1 in endothelial cells, alveolar macrophages, epithelial cells, and interstitial fibroblasts [381], contributing to pulmonary fibrosis. Chemotherapeutic drugs commonly used for treating ovarian and cervical cancer, induce a TGF-β gene signature, with an elevated expression of TGF-β1 [382]. In a study using two cervical and two ovarian cell lines, investigators found that the commonly used chemotherapeutic drugs, cisplatin, paclitaxel, doxorubicin, and camptothecin induced the expression of TGF-β1 mRNA and protein and induced the activation of Smad2 using a phospho-Smad2 antibody both by Western blot and by immunohistochemistry analysis [382]. They then used two different TGF-β inhibitors with which they enhanced the therapeutic effectiveness of cisplatin in mouse studies.

### 12.3. Role of TGF-β in the Mechanism of Resistance to Cancer Chemotherapeutic Agents

The acquisition of chemotherapy-resistant metastatic disease is a common occurrence in patients with triple-negative breast cancer (TNBC), attributed to chemotherapy-resistant CSCs. TGF-βs contribute to the development of CSCs in TNBC [288], thus supporting TGF-β inhibition as a strategy to reverse such chemotherapy resistance. An analysis of RNA expression in pre- and post-chemotherapy breast cancer biopsies revealed an increased expression of genes associated with CSCs and TGF-β signaling [383]. That study also showed that chemotherapy with paclitaxel enhanced TGF-β signaling, IL-8 production, and the growth of CSCs in TNBC cell lines and mouse xenografts. Moreover, the inhibition of TGF-β signaling by a TβRI kinase inhibitor Galunisertib or Smad4 siRNA effectively blocked paclitaxel-induced IL-8 transcription and CSC expansion. Furthermore, treatment with Galunisertib also prevented tumor reestablishment in TNBC xenografts following paclitaxel administration. These findings support that chemotherapy-activated TGF-β signaling promotes tumor relapse by expanding CSCs in an IL-8-contingent mechanism, and TGF-β pathway inhibitors could potentially circumvent the acquisition of drug-resistant CSCs. The investigators of that study advocated for testing a combination of chemotherapy with TGF-β blockade therapeutics in TNBC patients. 

The upregulation of TβRII has been identified as a common mechanism of acquired resistance against multiple anti-cancer drugs, including chemotherapeutics and molecular targeted therapies [289]. Importantly, the TβRI kinase inhibitor, LY2157299, reversed chemotherapy drug tolerance. These findings suggest that targeting TGF-β signaling could be a promising strategy to overcome drug resistance in cancer treatment.

### 12.4. Potential Role of TGF-β-Blockade Drugs in Immune Checkpoint Therapy 

The breakthrough in understanding immune checkpoints and the subsequent advancement of drugs, particularly monoclonal antibodies targeting programmed cell death protein 1/programmed cell death ligand 1 (PD-1/PD-L1), represents a pivotal moment in the landscape of cancer immunotherapy [384]. These treatments have exhibited robust and sustained antitumor effects across a spectrum of cancer types [385,386,387]. However, the challenge of a notably small response rate remains a significant bottleneck for anti-PD-1/PD-L1 therapies, aggravated by the absence of precise molecular markers for patient selection [388,389,390]. The PD-1/PD-L1 axis within the tumor does not singularly govern immunosuppression; other pathways contribute, with hyperactive TGF-β signaling in the TME emerging as a key player. This TGF-β signaling not only modulates diverse immune cell activities but also reshapes the TME, collectively fostering immune escape by tumor cells [391]. Crucially, the TGF-β and PD-1/PD-L1 pathways operate independently yet they may complement each other. Recent investigations have spotlighted TGF-β as a predictive factor for anti-PD-1/PD-L1 therapies, offering insights into treatment efficacy [392,393]. Consequently, the pursuit of TGF-β-involved predictive biomarkers and the exploration of TGF-β-targeted therapies stand as invaluable avenues for advancing cancer immunotherapy.

### 12.5. Translation of Preclinical Results into Clinical Success 

Notably, it is imperative to recognize that the efficacy observed in preclinical models with TGF-β inhibitors may not necessarily translate into clinical success, owing to factors beyond species differences. A significant discordance between preclinical and clinical outcomes in cancer therapy lies in the timing of treatment. In preclinical studies, therapeutics are typically administered shortly after animals are inoculated with tumor cells. In contrast, clinical treatment initiates when tumors have reached an advanced and highly aggressive stage. This difference in timing results in less effective drug penetration in clinical settings compared to preclinical scenarios. Moreover, patient enrollment in clinical trials frequently focuses on individuals with very advanced-stage cancers, where conventional treatments have already been exhausted. This approach inadvertently overlooks potential opportunities to utilize those inhibitors for the treatment of early-stage cancers. Given the demonstrated role of TGF-β signaling in the acquisition of chemotherapeutic drug resistance, it is also prudent to investigate whether such resistance is due to TGF-β responses, opening new opportunities to counteract drug resistance by TGF-β signaling blockade. The narrow therapeutic window of TGF-β inhibitor in human studies poses a substantially greater level of difficulty over that in mouse studies. In contrast to the uniformity of the population in mouse studies, assessing the appropriate dose of a TGF-β antagonist to work within a therapeutic window in clinical trials is challenging due to patient-to-patient differences in pharmacokinetic and pharmacodynamic parameters. Understanding and addressing these nuances are vital for translating preclinical observations into clinical practice.

### 12.6. TGF-β Receptor Kinase Inhibitors

**Figure 8:**
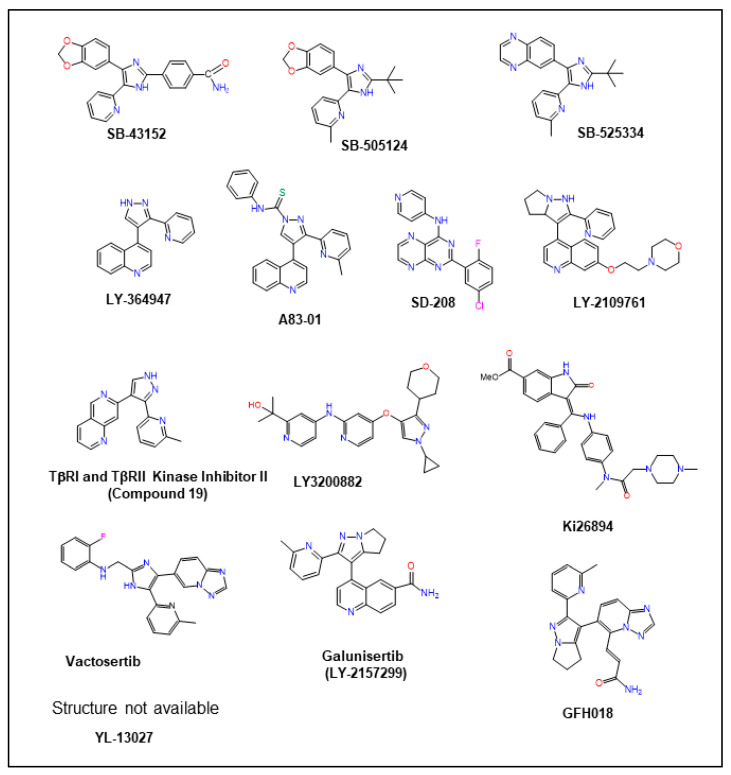
Chemical structures of various ALK5 kinase (TβRI) inhibitors used in preclinical and clinical studies.

Many selective TβRI kinase inhibitors have been developed for both preclinical and clinical applications (Figure 8). They include SB-431542 (first introduced in 2002) [394], SB-505124 (first introduced in 2004) [395], compound 19 (discovered in 2004) [396] SB-525334 (first reported in 2005) [397], Ki26894 (developed in 2007) [398], A83-01 (first reported in 2005) [399], SD-208 (first introduced in 2004) [400], LY364947 (also named HTS466284 or SM305, first reported in 2006), LY2109761 (TβRI and TβRII kinase inhibitor first reported in 2008) [401], LY3200882 (developed in 2020) [402,403], Vactosertib (EW-7197; developed in 2014) [404], LY2157299 (Galunisertib, developed in 2008) [405], GFH018 reported in 2019 [406], and YL-13027 reported in 2021 [407]. Despite their clinical efficacies and many benefits including oral bioavailability/accessibility/tumor penetration and relatively low production cost, these kinase inhibitors generally have non-specific or off-target effects (i.e., inhibit other kinases), relatively short half-lives, and comparatively narrow therapeutic windows. Because they target TGF-β receptors, they block the activity of all TGF-β isoforms equally. While their short half-lives may be a desirable trait due to a preference for intermittent drug dosing and the cessation of drug action following an adverse reaction, their low therapeutic windows have made achieving a non-toxic therapeutic dose challenging, given potential individual patient differences in pharmacokinetic parameters such as drug bioavailability, metabolism, and clearance, as well as potential pharmacokinetic and pharmacodynamic drug–drug interactions. 

SB-431542: The first reported TβRI kinase inhibitor, named SB-431542, was discovered and characterized in 2002 [394,408]. SB-431542 was developed by a series of chemical optimizations of a hit compound identified following the screening of a small chemical library with an ALK5 kinase assay, using GST-Smad3 as substrate [408]. SB-431542 acts as a competitive ATP binding site inhibitor of the TβRI kinase, and in various cell lines, between 0.1 μM and 5 μM of this compound effectively inhibits (>80%) the phosphorylation of Smad3. Besides inhibiting TβRI, also called the activin receptor-like kinase 5 (ALK5), SB-431542 also inhibited the activin type I receptor ALK4 and the nodal type receptor ALK7. Impressively, 10 μM SB-431542 did not inhibit other receptors in the TGF-β superfamily, namely the BMP type I receptors, ALK1 ALK2, ALK3, and ALK6. SB-431542 (10 μM) also did not inhibit numerous other kinases tested, including ERK, JNK, AMPK, GSK3β, MAPKs, p70S6K, PDK1, PKA, PKB, and PKCs. In its second application, 1 μM SB-431542 effectively blocked the induction of collagen IV and VEGF expression in mouse podocytes by either high glucose (25 mM, 14 days) or TGF-β1 (2 ng/mL, 24 h). 

SD-208: The characterization and use of the first orally active ALK5 kinase inhibitor (SD-208) with a specific activity and specificity similar to that of SB-43152, was first reported in 2004 [400]. This study also evaluated the effect of SD-208 on human LN-308 and murine SMA-560 glioma cells in vitro and SMA-560 gliomas implanted orthopedically in syngeneic mice. SD-208 effectively inhibited TGF-β-induced growth inhibition, migration, and invasion in glioma cells in vitro. SD-208, administered orally to mice in their water feed (at 1 mg/mL) extended the median survival of mice bearing gliomas. Histological analysis of tumors showed that SD-208 increased the tumor infiltration of NK cells, CD8 T cells, and macrophages without significant changes in blood vessel formation, proliferation, or apoptosis. These results supported a potential therapeutic benefit of SD-208 for treating malignant glioma and other malignancies.

**Table 1:** List of TGF-β Signaling Inhibitors Used in Preclinical and Clinical Studies.

Name	Target	Class	Pre-Clinical (Ref)	Clinical Phase/(Ref)	NCT Registry # (Drug Combination)	Cancer Types/Indications (Pt Numbers)	Efficacy	Most Frequent Adverse Events (AE)
**SB-431542**	TβRI kinase	SCI	[394]	None		Various cell lines; mouse studies	0.1–5 μM > p-Smad3 Inhibits ALKs 5, 4 and 7, but not ALKs 1, 2, 3, and 6 and many other kinases.	No signs of toxicity in mice [409].
**SB-505124**	TβRI kinase	SCI	[395]	None		Various cell lines; mouse studies	3- to 5-fold greater potency than SB-431542, similar specificity to SB-431542; 76.4% oral bioavailability.	Tx was not adequately assessed.
**SB-525334**	TβRI kinase	SCI	[397]	None			IC50 of 58.5 nM on ALK5, >200 nM on ALK4; effective in mice orally at 1 to 10 mg/kg/d.	No significant toxicity on human peritoneal mesothelial cells up to 10 μM [410].
**Ki26894**	TβRI kinase	SCI	[398]	None				Tx was not adequately assessed.
**A83-01**	TβRI kinase	SCI	[399]	None				
**SD-208**	TβRI kinase	SCI	[400]	None		Human and murine glioma cells	Inhibited TGF-β-induced growth inhibition, migration, and invasion of glioma cells; extended survival of mice bearing glioma.	Well tolerated, without observable Tx.
**LY364947, (HTS466284, SM305)**	TβRI kinase	SCI		None				Tx was not adequately assessed.
**LY2109761**	TβRI and TβRII kinases	SCI	[401]	None		Orthotopic murine models of PC	Effective in inhibiting PC growth in combination with gemcitabine; prolonged mice survival.	Tx was not adequately assessed.
**LY3200882**	TβRI kinase	SCI	[402]					
				[403] Phase I	NCT02937272 Single-agent ± anti-cancer agents (gemcitabine, paclitaxel)	Advanced cancers (139 pts): glioma, PC, HNSCC	Durable PP in Glioma. 75% DCR in the combination arm of PC pts.	39.6% AE; grade 3 only in combination arm; rare CV toxicity (one in 139 pts).
**Vactosertib** **(EW-7197)**	TβRI kinase	SCI	[404]				IC50 = 12.1 to 16.5 nM on ALK5 and ALK4. Did not inhibit 320 other kinases tested. Inhibitor of fibrosis in animal models.	
			[411]			4T1 BC	Inhibited cell migration, invasion, and lung metastasis.	Optimal dosing not established.
			[412]			Osteosarcoma	Tumor regression, blocked tumor invasion, and prolonged survival [413].	No severe toxicity [404,411].
				[414] Phase I	NCT02160106 Dose escalation. 30 mg–340 mg/d for 5 d, 2 d off.	PC (29 pts)	T_1/2_ = 3.2 h 6 pts of 16 treated pts achieved stable disease at ≥ 140 mg/d	Excellent overall safety. Most common: fatigue. One pt of 16 pts had abdominal pain, pulmonary edema, and liver enzyme elevation. One pt with stroke at 100 mg/d.
**Galunisertib** **(LY2157299)**	TβRI kinase	SCI		Phase II [415]	NCT01246986 (+ Sorafenib) (47 pts)	Advanced HHC	Prolonged OS (18.8 m), PSF (4.1 m), and PR in 2 pts, SD in 2 pts.	Acceptable safety profile. 1 pt grade 4 renal injury. Diarrhea (43.2%) and pruritis (25%). 59.6% pts with a serious EV.
				Phase II [416]	NCT02008318 (41 pts)	MDS	Hematologic improvements in 24.4% pts; 44% pts had reduced fatigue.	Acceptable safety profile. EV included fatigue (20%), diarrhea (17%), pyrexia (12%), and vomiting (12%).
				Phase II [417]	NCT01220271 (+ Temozolomide-radiation therapy) (56 pts)	Malignant gliomas	Improved DRR (80%).	Fatigue, nausea, and constipation.
				Phase II [418]	NCT02688712 (+ Radiotherapy) (38 pts)	Localized CRC	Complete response in 38% of pt at 1 yr.	Grade 3 EV (diarrhea in 16%, hematological Tx in 18%). Two pts had grade 4 EV related to radiotherapy and ischemia.
				Phase II [419]	NCT02734160 (+ Durvalumab) (32 pts)	Metastatic PC	1 pt PR, 7 pts SD, and 15 pts had objective progressive disease. 25% DCR. Median OS (5.72 m), and PRS (1.87 m).	No dose-limiting toxicity was recorded.
				Phase II [420]	NCT01246986 (+ Ramucirumab) (8 pts)	Advanced HCC	MTD was established at 150 mg/d/twice daily with 8 mg/kg ramucirumab every 2 wks.	No dose-limiting Tx was observed. EV included nausea in 25% pts, and vomiting in 25% pts. One pt cerebrovascular accident.
**GFH018**	TβRI kinase	SCI		[406] Phase I	NCT05051241	ASTS (50 pts)	MTD = mg BID, 14 d Stable disease (9 pts), tumor shrinkage (1 pt).	Mostly Grade 1 and 2, proteinuria, anemia, and increased liver enzymes.
**YL-13027**	TβRI kinase	SCI		[407] Phase I	NCT03869632 60–300 mg/day	ASTS (13 pts)	MTD not reached T_1/2_ = 4.2 h	Anemia, + GGT.
**Long-Acting Tumor-Activated Prodrug**	TβRI kinase	SC	[421]	None			Long-acting.	No mortality in tox studies. Valvulopathy (50% rats).
								
**Fresolimumab (GC-1008**	TGF-β1, TGF-β2, TGF-β3	mAb		[422] Phase I (29 pt)	NCT00356460 Dose escalation 0.01 to 15 mg/kg every 2 wks	RCC (28 pts), MM (1 pt).	1 pt PR, 6 pts SD, 24 wks PFS, no DLT up to 15 mg/kg. T_1/2_ = 21.7 d	Hyperkeratosis, non-malignant keratoacanthomas at high drug doses
				[423] Phase II	NCT01401062 (+ radiotherapy) (23 pts)	Metastatic BC	Longer mean survival and improved PMC count at 10 mg/kg than at 1 mg/kg	Well tolerated; 7 grade ¾ AE in 5 of 11 pts in 1 mg arm and 2 of 12 in 10 mg arm.
				Phase II	NCT00923169	Advanced MM	Results pending.	
				Phase II	NCT01291784	MF	Results pending.	
**TβM1 (LY2382770)**	TGF-β1	mAb		[424]		Advanced MM (18 pts), 20 to 240 mg/m	T_1/2_ = 9 days SD; no significant response; discontinued.	Generally safe; nausea, diarrhea, & fatigue in 15% pts.
**NIS793**	TGF-β1, TGF-β2, TGF-β3	mAb		[425]	NCT02947165 + Spartalizumab	AST (60 pts), MSS-CRC, anti-PD1-resistant NSCLC	Target engagement and TGF-β inhibition.	No DLT up to 30 mg/kg NIS797 +300 mg/kg Spartalizumab every 3 wks.
**SAR439459**	TGF-β1, TGF-β2, TGF-β3	mAb	[426]		NCT03192345	Analysis of 1000 pts’ tumors	Achieved significant correlation of high TGF-β pathway with resistance to anti-PD-1.	Not applicable.
				[427] Phase I	±Cemiplimab	AST (52 pts)	Reduced plasma TGF-β1; induced immune cell activation.	DLT observed, MTD not achieved Acceptable tolerability profile.
**XPA-42-089**	TGF-β1, TGF-β2, TGF-β3	mAb	[428]	None		±anti-DP-1 in SSC syngenic mice	10–20% complete tumor regression.	
**Pan-TGFβ mAb**	TGF-β1, TGF-β2, TGF-β3	mAb	[102]	None			Toxicology studies in mice and monkeys.	Significant toxicities: systemic bleeding, CV effects after 5 weeks IV administration of 30 or 100 mg/kg.
**IMC-TR1 (LY3022859)**	TβRII	mAb		[429] Phase I	NCT01646203	Standard chemotherapy-resistant ASD (14 pt)	Primary objective of safe effective dose not achieved.	Cytokine syndrome, infusion-related reactions.
**Anti-LAP**	TGF-β1 LAP	mAb	[430]	None		Mouse models of MM, CRC, GBM	10 mg/kg every 3 days decreased tumor growth, LAP +Treg and tolerogenic DC.	Not assessed.
**SRK-181**	Latent TGF-β1	mAb	[431]			Syngeneic mouse models of UC, MM, BC	SRK-181 + anti-PD-1 mAb induced robust antitumor responses, and improved survival of animals bearing anti-PD1-resistant tumors. Restored sensitivity to anti-PD-1 mAb.	No CV Tx.
					4-week Tx study in rats and monkeys.			Well tolerated, no treatment AE at 200 mg/kg in rats and 300 mg/kg in monkeys.
				Phase I	NCT04291079 ± anti-PD-L)1	ASTS	No results yet.	No results yet.
**ABBV-151**	GARP	mAb		[432]	NCT03821935 + Budigalima (248 Pt)	Locally advanced or metastatic solid tumors	Enhanced response in anti-DP-1-resistant UC. ORR = 10%	17% pts ≥ grade 3 AE.
						HCC		Safety concerns—discontinued.
**PIIO-1**	GARP	mAb	[407]			Murine cancer models	Reduced thrombocytopenia, enhanced CD8+ T cells function, reduced TGF-β signaling.	
**DS-1055a**	GARP	mAb	[433]			HT-29 CRC in humanized mice	Robustly blocked GARP in the TME, suppressed tumor growth.	
**C6D4**	αVβ8	mAb	[434]				C6D4 (10 mg/kg, once to twice weekly) can significantly reduce tumor growth and improve survival.	
**ADWA-11**	αVβ8	mAb	[435]			SCC, BC, CRC, and PCa in syngeneic mice ± radiotherapy ± immunotherapy	Suppression or complete regression of tumor growth; enhanced expression of gene linked to cell tumor killing in CD8+ T cells.	
								
**AVID200 (Fc-TβRII)**	TGF-β1, TGF-β3	Ligand traps		[436] Phase I	NCT03895112	MF (21 pts)	Two pts met clinical benefit with improvement of symptoms; improvement of platelet counts in 81% of pts. Two patients attained clinical benefit with spleen and symptom improvement.	No DLT. Grade 3/4 anemia and thrombocytopenia in a subset.
**4T-Trap**	TGF-β1, TGF-β3, CD4	Ligand trap-mAb bifunctional protein	[437]				Twice weekly IV administration inhibits Th cell TGF-β signaling in CD+ lymph nodes. Improved tumor killing.	Induced tumor hypoxia.
**Bintrafusp Alfa (M7824)**	TGF-β1, TGF-β3, PD-1	Ligand trap-mAb bifunctional protein	[438,439]			A range of human cancers.	Reduce Treg on human CD4+ T-cell proliferation.	
				Phase I [440]	NCT02517398	NSCLC (80 pts) with disease progression after platinum-based therapy. Pt were randomized to receive 500 mg/d or 1200 mg/d every 4 wk.	ORR = 21.3% at 500 mg dose. Tumors with higher PD-L1 levels showed higher response rates.	Treatment-related AE in 69% pt; 29% pts grade 3 or higher AE; 10% pt discontinue treatment; no treatment-related deaths.
				Phase I [441,442]	NCT02699515	Advanced gastric/gastroesophageal junction cancer 1200 mg/2 wks (31 pts)	ORR = 16%; DCR = 26%	19% treatment-related grade 3 AE; no grade 4 EA. 19% immune-related EA.
				Phase I [443]	NCT02517398	SCCHN (32 pts)	ORR = 13%; PR = 29% pt; DCC = 34% pt.	23% pt grade 3 AE; grade 3 treatment-related AE = 34% pt. No grade 4 AE or treatment-related death.
				Phase III [444]	NCT03631706	PD-L1-high advanced NSCLC (304 pts) received Bintrafusp Alfa or pembrolizumab	No significant difference in endpoints was observed between treatment groups.	About 3-fold more grade 3–4 AV in the Bintrafusp Alfa group than in the pembrolizumab group. The study was discontinued.
**SHR-1701**	TGF-β1, TGF-β3, PD-1	Ligand trap-mAb bifunctional protein		Phase I	NCT03774979	Recurrent or metastatic CC following platinum-based therapy (32 pt)	ORR = 15.6%, ongoing response in 80% of responders, DCR = 50%.	Treatment-related EA of grade 3 or 4 in 34% pts. No treatment-related deaths.
				Phase I	NCT03710265	ASTS (171 pt)	20% ORR 54.5% OSR	No DLT observed.
**YM101**	TGF-β1, TGF-β2, TGF-β3, PD-L1	Bispecific mAb	[445]	None		BC, CRC, murine T cells in syngeneic mice	Counteract the biological effects of TGF-β and PD-1/PD-L1 pathways; superior antitumor activity compared to monotherapy by anti-TGF-β or anti-PD-1/PD-L1.	
**BiTP**	TGF-β1, TGF-β2, TGF-β3, PD-L1	Bispecific mAb	[446]	None		TNBC in syngeneic mice	Similarly effective compared to YM101; enhanced immune cell penetration by reducing collagen deposition.	
								
**Trx-SARA**	SARA	Peptide Aptamer	[447]	None		NMuMG murine mammary epithelial cells	Binds to Smads 2 and 3, inhibits TGF-β responses.	Not assessed.
**APT-β1**	Active TGF-β1	Nucleotide Aptamer	[448]	None		NSCLC xenografts in mice (±gefitinib)	Enhanced effectiveness of gefitinib on tumor regression. More potent than TGF-β1 mAb.	Not assessed.
**Aptamer S58**	TβRII extracellular domain	Nucleotide Aptamer	[449]	None		Human tendon fibroblasts	Inhibited aSMA expression and incorporation into stress fibers.	Not assessed.
**Trabedersen (AP12009)**	TGF-β2	ASO		[450] Phase I	NCT00844064	CRC, PC, MM		
				[451] Phase II	NCT00431561, NCT00761280	Refractory AA or secondary GBM (145 pts)	19 pts CRR or PR, improved OS of responders.	Nervous disorders.
				Phase IIb	NCT05935774 + atezolizumab	Metastatic or recurrent NSCLC	Study withdrawn.	
**ISTH0036**	TGF-β2	ASO		[452] Phase I	NCT02406833	Glaucoma patients, intravitreal injection	Likely effective.	Likely safe.
**AP11014**	TGF-β1	ASO	[453]			PCa, CRC, NSCLC	Meeting abstract only, 2004.	Meeting abstract only, 2004.
**ISTH0047**	TGF-β2	ASO	[454]			Glioma	Inhibited TGF-β2 and growth and invasion of glioma cells, prolonged host survival. Not a well-controlled study.	Not adequately assessed.
**ISTH10047**	TGF-β1	ASO	[454]			Glioma	Inhibited TGF-β2 and growth and invasion of glioma cells, prolonged host survival. Not a well-controlled study.	Not adequately assessed.

Abbreviations: AA (anaplastic astrocytoma), AE (adverse events), ALP (alkaline phosphatase), ASTS (advanced solid tumors), SCI (small chemical inhibitor), BC (breast cancer), BID (twice daily), CC (cervical cancer), CRR (complete response rate), CRC (colorectal cancer), CV (cardiovascular), DC (dendritic cells), DCR (disease control rate), DLT (dose-limiting toxicity), d (day) GBM (glioblastoma), GGT (γ-glutamyltransferase), HCC (hepatocellular carcinoma), HNSCC (head and neck squamous cell carcinoma), MDS (myelodysplastic syndromes), PCa (prostate cancer), MF (myelofibrosis), MC (metastatic cancers), mon (months), MSS (microsatellite stable), NSCLC (non-small cell lung cancer), OS (overall survival), OSR (overall survival rate), PC (pancreatic cancer), PFS (progression-free survival), PR (partial response), pt (patient), Ref (reference), (Tx (toxicity), T_1/2_ (half-life), SD (stable disease), SCC (squamous cell carcinoma), UC (urothelial cancer), wk (week), yr (year).

The therapeutic potential of the oral administration of SD-208 (20 mg/kg/day or 60 mg/kg/day) was further explored on mammary tumor growth and metastasis in vivo using R3T and 4T1 cells inoculated in the fat pad of syngeneic mice [455]. SD-208 hampered primary tumor growth and significantly reduced the size and number of lung metastases in a dose-dependent manner. The antitumor effects were observed in different mammary carcinoma models and were specific to syngeneic mice as SD-208 did not inhibit the growth of R3T tumors in athymic mice. The study also explored the pharmacokinetic and pharmacodynamic properties of SD-208. Although plasma levels of SD-208 varied among different mouse strains, SD-208 was well-tolerated during continuous administration without observable toxicity. SD-208 suppressed the level of pSmad2 levels along with the expression of several TGF-β-regulated genes in tumor tissue and increased tumor-specific CTL and eosinophil infiltration. In conclusion, in this study SD-208 demonstrated potent antitumor effects in vivo, suppressing both primary tumor growth and metastases, likely through the TGF-β modulation of gene expression, and immune response.

In myeloma patients, bone disease is common and debilitating, leading to increased fractures and mortality. In a mouse model of aggressive multiple myeloma (JJN3), SD-208 given by oral gavage (60 mg/kg for two weeks) in combination with chemotherapy (Bortezomib + Lenalidomide) more effectively prevented bone lesions and improved bone quality in immunocompromised mice bearing JJN3 xenografts than did chemotherapy alone [456]. SD-208 was superior to the TGF-β monoclonal antibody ID11, administered by i.p. injection in preventing bone lesions in this model. SD-208 did not affect mineralization but improved collagen matrix maturation, contributing to enhanced mechanical strength. Overall, this study supported that targeting TGF-β with SD-208, in combination with chemotherapy, holds promise for treating and preventing myeloma bone disease. SB-208 has been used with therapeutic success in preclinical models of prostate cancer [457] myeloid leukemia [458], neurofibromatosis [459], PC [460,461], and melanoma [462]. However, SD-208 failed to inhibit the in vivo tumor growth of the human colon cancer cell model SW-48 [463]. Despite its overall favorable response, SD-208 has not entered clinical development. 

SB-505124, introduced in 2004, was discovered as a small molecule kinase inhibitor of TβRI with structural features of SB-43152, but with 3- to 5-fold greater potency [395]. Similar to SB-43152, SB-505134 blocked the activation of Smad2 and Smad3 and mitogen-activated protein kinase pathway components (p38-MAPK, ERK1/2, and JNK) induced by TGF-β1 or ALK4, ALK5, and ALK7, and blocked cell death induced by TGF-β1. As expected, SB-505124 failed to block the activation of ERK1/2 or p38-MAPK by EGF. Moreover, SB-505124 does not affect signaling by other type I receptors in the TGF-β superfamily, namely ALK1, ALK2, ALK3, or ALK6. A pharmacokinetic study using a validated LC-MS/MS method showed a 76.4% bioavailability of SB-505124 in rats following its oral administration (10 mg/kg) [464]. Since its discovery, SB-505124 has been employed in numerous cell and mouse models to interrogate TGF-β signaling in normal and disease states. While it shows good therapeutic promise, it has not yet entered clinical development.

SB-525334, introduced in 2005, was discovered as a potent (58.5 nM) and selective inhibitor of ALK5 kinase, with 4-fold lower potency on ALK4 and inactive on ALKs 2, 3, and 6 (>10 μM) [397]. Oral administration (1 to 10 mg/kg/day for 11 days) of SB-525334 in a mouse study effectively suppressed TGF-β responses, particularly the expression of PAI-1, collagen I, and collagen III mRNAs, in nephritic kidneys induced by puromycin. Since its discovery, SB-525334 found its usefulness in 48 other publications studying TGF-β responses in cell culture and mouse models of cancer and fibrosis but as of yet has not been used clinically. 

LY2109761, the first discovered TβRI and TβRII dual kinase inhibitor [401], exhibiting high potency and specificity compared to other ALK5 inhibitors and oral bioavailability, was used successfully in an orthoptic murine model of PC. In combination with Gemcitabine, LY2109761 substantially decreased the tumor load of PC cells and prolonged the overall survival (OS) of bearing pancreatic tumors. It also inhibited abdominal metastases. LY2109761 has been used in numerous other mouse studies using various preclinical cancer models, resulting in 140 publications to date. It has not entered clinical testing.

LY3200882: The selective ALK5 kinase inhibitor, LY3200882, which was developed in 2020 [402] was quickly introduced in a phase I clinical trial in 2021 [403] based on highly promising preclinical data. The first-in-human trial of LY3200882 comprised a multicenter study with 139 patients enrolled (NCT02937272). The study’s primary objectives included assessing the safety, tolerability, pharmacodynamics, pharmacokinetics, and preliminary antitumor action of LY3200882, either as a standalone therapy or in conjunction with other anticancer agents, in patients with advanced cancers. The trial encompassed dose escalation, monotherapy expansion in grade 4 glioma, and combination therapy in solid tumors, pancreatic cancer, and head and neck squamous cell cancer. Out of the 139 treated patients, the majority experienced treatment-emergent adverse events, with 39.6% of patients having LY3200882-related events. Notably, grade 3 LY3200882-related toxicities were observed only in combination therapy arms. Cardiovascular toxicity was reported only in one patient in the PC arm. The study established LY3200882 monotherapy recommended phase II doses in two schedules (35 mg or 50 mg twice daily 2 weeks on/2 weeks off). Durable partial responses were observed in patients with grade 4 glioma, and in treatment-naïve patients with advanced PC, the combined treatment of gemcitabine and paclitaxel with LY3200882 showed a remarkable 75% disease-control rate. The findings suggest that LY3200882, both as monotherapy and in combination with chemotherapy, is safe, well-tolerated, and shows promising antitumor activity in PC, warranting further studies to evaluate its efficacy in advanced cancers.

Galunisertib (LY2157299), which was first developed in 2008, is a small molecule inhibitor of ALK5 kinase, and has been shown to inhibit tumor growth in preclinical and clinical studies. It has been studied in combination with chemotherapy and immunotherapy in clinical trials for several types of cancers, including PC [419], hepatocellular carcinoma [415], esophageal cancer [465], and GBM [417]. The therapeutic window of Galunisertib was first assessed through a pharmacokinetic/pharmacodynamic model in which simulations were conducted to assess population plasma exposures and biomarker responses in GBM and other tumors to Galunisertib [377]. This study predicted a therapeutic window between 160 and 360 mg of Galunisertib, based on a 30% inhibition of ALK5 kinase. The study defined a safe range for using Galunisertib in cancer patients, using a model that considers how the drug works and its safety in early cancer treatment stages. Galunisertib phase I and II trials involved vigilant examination for adverse cardiac events [374]. Safety studies concluded that Galunisertib was relatively safe in humans, leading to the resumption of clinical trials with intermittent dosing (2 weeks on/2 weeks off). A phase 2 clinical trial (NCT01246986) [415] next explored the combination of Galunisertib and Sorafenib, revealing acceptable safety profiles and prolonged OS, advocating for further evaluation in larger trials. Valeria Santini et al. (NCT02008318) [416] also conducted a phase II trial of Galunisertib in intermediate- to low-risk myelodysplastic syndromes, demonstrating hematologic improvements with an acceptable safety profile, highlighting potential applications in hematological malignancies. 

In 2020, Antje Wick et al. (NCT01220271) [417] investigated Galunisertib in combination with temozolomide-based radiochemotherapy for malignant glioma, reporting comparable efficacy and safety, further supporting the feasibility of TGF-β inhibition in combination strategies. Galunisertib was tested on CRC in a phase 2 study by Tomoko Yamazaki et al. in 2022 (NCT02688712) [418], and demonstrated improved complete response rates in locally advanced rectal cancer in combination with neoadjuvant chemoradiotherapy. In a different context, in 2021 Davide Melisi et al. (NCT02734160) [419] assessed the safety and activity of Galunisertib plus Durvalumab in metastatic PC, exploring the potential synergistic effects of combining TGF-β inhibition with immune checkpoint blockade. Harding et al. [420] (NCT01246986) conducted a phase 1b study investigating the combination of Galunisertib and Ramucirumab in patients with advanced hepatocellular carcinoma, establishing the MTD and safety profile, providing insights into the interplay between VEGF and TGF-β signaling.

Vactosertib (EW-7197) was developed and characterized in 2014 [404] as a highly potent and bioavailable kinase inhibitor of TβRI, with potent biological effects (IC50 = 12.1 to 16.5 nM) inhibiting responses in various cancer cell line models. Vactosertib selectively inhibited ALK5 and ALK4 but did not inhibit any kinase in a panel of 320 kinases, supporting high specificity for the TβRI kinases. Soon after, Vactosertib’s anti-fibrotic potential was assessed in various mouse and rat models, including CCl4-induced liver injury, bile duct ligation, bleomycin-induced lung fibrosis, and unilateral ureteral obstruction-induced kidney fibrosis [466]. Vactosernib demonstrated efficacy by reducing collagen, α-smooth muscle actin, fibronectin, 4-hydroxy-2, 3-nonenal, and the expression of integrins in respective organs. In vivo studies revealed that Vactosertib extended the lifespan of animals treated with the above fibrotic inducers. Mechanistically, Vactosertib inhibited fibrosis by TGF-β1/Smad2/3 and ROS signaling. In another study, Vactosernib demonstrated significant antimetastatic efficacy in a mouse model of breast cancer (4T1), inhibiting Smad/TGFβ signaling, cell migration, invasion, and lung metastasis [411]. Additionally, it suppressed EMT and enhanced cytotoxic T lymphocyte activity, leading to increased survival in breast tumor-bearing mice. Vactosernib also promoted the regression of osteosarcoma in a pre-clinical mouse model [412] and inhibited the invasion of pancreatic tumors along with prolonging the survival of mice bearing such tumors [413]. Overall, Vactosernib exhibited potent in vivo antimetastatic activity, suggesting its potential as a cancer therapeutic. 

A first-in-human trial published in 2020 (NCT02160106) [414] investigated the pharmacokinetics of Vactosertib in patients with advanced solid tumors. Data from 29 patients revealed a rapid absorption and elimination of Vactosertib, with a terminal median half-life (T1/2) of 3.2 h and median time to a maximum concentration of 1.2 h. The pharmacokinetics appear dose-proportional within the tested range, with negligible accumulation after five days of once-daily dosing. However, considering the short half-life, the study suggests the need for twice- or thrice-daily administration to maintain effective concentrations. While the study provides valuable insights into Vactosertib’s pharmacokinetics, further research is warranted to establish optimal dosing strategies for clinical applications. Vactosertib is currently under clinical trial investigation as a monotherapy for metastatic ductal carcinoma (NCT04258072), and refractory osteosarcoma (NCT05588648), and in combination therapy with paclitaxel and Ramucirumab for metastatic gastric adenocarcinoma (NCT04656002), with Pomalidomide for relapsed and refractory multiple myeloma (NCT03143985), with Pembrolizumab for melanoma (NCT05436990), with Imatinib for Desmoid tumors (NCT06219733), with Durvalumab for urothelial carcinoma (NCT04064190), with Durvalumab in gastric cancer (NCT04893252), with Pembrolizumab in colorectal and gastric cancer (NCT03724851), with Pembrolizumab for CRC and hepatic metastases (NCT03844750), with Paclitaxel for metastatic gastric cancer (NCT03698825), with chemotherapy for esophageal adenocarcinoma (NCT06044311), and with Pembrolixumab for PD-L1 positive non-small cell lung cancer (NSCLC) (NCT04515979).

GFH018: A phase I clinical trial investigated the safety, pharmacokinetics, and preliminary efficacy of GFH018, a TβRI kinase inhibitor, in advanced solid tumor patients (NCT05051241) [406]. Conducted with 50 enrolled patients, the results reveal a favorable safety profile and preliminary anti-tumor activity of GFH018, establishing a maximum tolerated dose (MTD) of 85 mg BID, 14 days on/14 days off. Adverse events, predominantly grade 1 or 2, included proteinuria, liver enzyme increases, and anemia. Nine patients achieved stable disease, with one experiencing tumor shrinkage. Despite limitations like small sample size and the absence of a control group, the study suggests the potential efficacy of GFH018 in advanced solid tumors, supporting ongoing combination studies with anti-PD-1 mAb Toripalimab and concurrent chemoradiotherapy. However, caution is warranted due to preliminary efficacy results from a limited patient cohort and assay issues affecting pharmacodynamic data, necessitating larger, controlled trials for a robust evaluation of GFH018’s clinical significance in this context.

YL-13027: Following the success of inhibiting tumors in mouse studies, YL-13027, a TβRI kinase inhibitor, entered a phase I trial (NCT03869632) [407] to characterize its safety, tolerability, and MTD in a cohort of patients with advanced solid tumors. Administered orally in escalating daily doses from 60 mg to 300 mg for at least two 28-day cycles, the drug demonstrated safety and tolerability in thirteen enrolled patients, with gastrointestinal, esophageal, gallbladder, lung, and breast carcinomas. No MTD was reached, and adverse events were manageable, including increased gamma-glutamyltransferase and decreased hemoglobin. Pharmacokinetic analysis revealed a rapid plasma concentration peak and a mean elimination half-life of 4.2 h. Of the six evaluable subjects, one with TNBC showed a partial response, indicating tumor reduction. The study concludes that YL-13027 is well-tolerated, supporting further clinical investigation. However, the limited sample size and the absence of a defined MTD could pose challenges in fully assessing its efficacy and safety profile. Additionally, long-term outcomes and broader patient cohorts are essential for a comprehensive evaluation of YL-13027’s potential in cancer treatment. YL-13027 is currently under/planned for clinical investigation for the treatment of advanced tumors (NCT05228600, NCT05457517), and in combination therapy with gemcitabine for metastatic PC (NCT06199466).

Long-Acting Tumor-Activated Prodrug of a TβRI Inhibitor: To increase the therapeutic window of TβRI kinase inhibitors, Zhang and colleagues recently [421] designed TβRI kinase inhibitor prodrugs preferentially activated in tumors over normal tissue based on cleavage and subsequent activation by proteases far more abundant in tumors than normal tissues. These prodrugs are highly potent and long-acting (treatment once weekly) compared to the short half-life of the parent compound small molecule inhibitors. While the most active prodrug appears to be more effective and less toxic than the parent compound and the lowest effective dose for 3 months did not cause mortality, about 50% of rats developed valvulopathy during toxicology studies.

### 12.7. Monoclonal Antibodies

Monoclonal antibodies (mAbs) have been used to treat patients for several decades. Notable milestones include the approval of Rituximab in 1997 for non-Hodgkin lymphoma, Trastuzumab in 1998 for breast cancer, and Infliximab in 1998 for autoimmune disorders [467]. In recent years, the application of mAbs has expanded significantly across various medical domains, including oncology, autoimmune diseases, and infectious diseases. More recent examples include the use of mAbs in cancer immunotherapy (checkpoint inhibitors) and the development of therapeutic antibodies for conditions such as rheumatoid arthritis and COVID-19. The major benefits of mAbs over small molecule inhibitors are their target-specificity, binding avidity (or specific activity), generally low toxicity, and their relatively long half-life (21–27 days), which is attributed to their Fc domain. Their very high specificities enable the design of highly specific reagents, selectively targeting a given TGF-β isoform, TGF-β receptor, and an extracellular protein involved in the activation of TGF-βs. They could readily be developed to recognize small epitopes (eight to ten amino acids) and 3-dimensional protein topology. However, as 150 kDa proteins, they have difficulty in penetrating tumor tissues, particularly desmoplastic ones with excess extracellular matrix, and certainly cannot be designed to readily penetrate cells to target intracellular epitopes. A major downside is their very high cost of production, leading to a high overall cost of patient therapy. Another downside is the potential to elicit an allergic reaction or anaphylaxis in some individuals. 

While mAbs exhibit superior specificity compared to small molecule inhibitors, their specificity is not absolute, potentially leading to off-target effects depending on the targeted epitope. Diverse neutralizing monoclonal antibodies can be developed against the same protein, binding to distinct epitopes and possessing varying properties such as affinity and specificity. These factors play a crucial role in accurately evaluating the efficacy and adverse effects of pan-specific versus monospecific TGF-β neutralizing antibodies. For instance, the safety profile of the pan-specific anti-TGF-β mAb Fresolimumab (332) contrasts with the significant adverse effects observed in another humanized pan-TGF-β mAb from Genentech [102]. Despite Fresolimumab demonstrating greater tumor response compared to the TGF-β1-specific mAb TβM1 [424], it remains challenging to definitively attribute the benefit to its ability to block all three TGF-β isoforms rather than just one. Recent studies utilizing the latent TGF-β1 mAb SRK-181, selectively blocking TGF-β1 without affecting TGF-β2 and TGF-β3, showed remarkable efficacy in regressing tumors in mouse studies without any signs of toxicity [64]. This highlights the importance of generating multiple antibodies against a specific target and subjecting them to rigorous testing before drawing conclusive insights about a therapeutic target versus its targeting agent. Overall, a comprehensive understanding of the distinct properties and effects of various monoclonal antibodies is essential for informed therapeutic decision-making.

Fresolimumab (GC-1008; human TGF-β mAb), which blocks the activities of three isoforms of TGF-β, entered clinical trials for advanced renal cell carcinoma, malignant melanoma, high-grade glioma, and radiation-treated metastatic breast cancer, showing significant tumor responses. In a multi-center phase I trial involving twenty-eight advanced metastatic melanomas and one renal cell carcinoma (NCT00356460) [422], Fresolimumab exhibited promising results. One patient attained a partial response, and six developed stable disease, resulting in a median 24-week progression-free survival. In that study, Fresolimumab had an acceptable safety profile, with no dose-limiting toxicities up to 15 mg/kg. The pharmacokinetics of Fresolimumab were linear and dose-proportional, with an overall half-life of 21.7 days. Notably, the development of treatment-emergent skin lesions, including hyperkeratosis and eruptive non-malignant keratoacanthomas (KA), was observed, seemingly associated with higher Fresolimumab exposure. However, these lesions spontaneously resolved over weeks to months, resembling non-malignant KAs rather than true SCCs. Nevertheless, the small sample size and potential bias in patient assignment limit firm conclusions. In a high radiation-treated metastatic breast cancer study, Fresolimumab was well tolerated and patients in the high-dose Fresolimumab group (10 mg/kg, once every 3 weeks) had a longer mean survival and a more favorable systemic immune response compared to the lower dose group (1 mg/kg, once every 3 weeks) (NCT01401062) [423]. Fresolimumab has also been used in other clinical trials for the treatment of advanced malignant melanoma (NCT00923169) and myelofibrosis (NCT01291784), with results pending.

TβM1 (LY2382770), a humanized TGF-β1-specific mAb: A dose escalation phase I clinical trial study of TβM1 was conducted with 18 patients in various types of advanced metastatic cancer [424]. Patients were treated with doses ranging from 20 to 240 mg per month (with doses based on preclinical results in mice), with the primary focus on safety, pharmacokinetics, and pharmacodynamics. The results indicated that the treatment was generally safe, with nausea, diarrhea, and fatigue being the most frequent side effects (observed in 17% of patients). However, treatment was discontinued after two to four cycles due to no noticeable benefit from TβM1. While TβM1 demonstrated good tolerability and an average half-life of 9 days, no significant pharmacodynamic effects were achieved, as evidenced by non-significant reductions in gene expressions and mixed results in tumor progression markers. The best clinical response observed was stable disease, and the lack of a consistent antitumor effect across various cancers raises questions about the drug’s clinical efficacy. Based on these results, further clinical development of TβM1 was discontinued. However, the study design had many limitations, such as small sample size, brief treatment duration, and inadequate pharmacodynamic response, underscoring the need for further research with larger cohorts and extended treatment periods to determine the potential utility of TβM1. Other limitations could be the heterogeneity of tumor types and the aggressiveness of cancers in this cohort. 

NIS793 (human pan anti-TGF-β mAb): A first-in-human trial (NCT02947165) [425] using a cohort of 60 patients explored the therapeutic potential of NIS793, a human pan anti-TGF-β mAb, in conjunction with the anti-PD-1 mAb Spartalizumab for treating advanced solid tumors. The investigation encompassed dose escalation and expansion phases, involving patients with microsatellite stable CRC (MSS-CRC) or anti-PD1-resistant NSCLC. The recommended dose of NIS793 was determined as 30 mg/kg and Spartalizumab 300 mg every 3 weeks, with manageable adverse events and no observed dose-limiting toxicities. The study’s strength lies in providing insights into the proof of mechanism for NIS793 through evidence of target engagement and TGF-β pathway inhibition, supported by biomarker and gene expression analyses. However, limitations, including a small sample size and lack of a control group, underscore the need for further research with larger cohorts and extended follow-up to validate findings and ascertain the clinical significance of NIS793 for the treatment of advanced solid tumors. NIS793 is under clinical investigation in combination therapy with standard-of-care chemotherapy (±Spartalizumab) for metastatic pancreatic ductal carcinoma (NCT04935359, NCT04390763) and colorectal carcinoma (NCT04952753).

SAR439459 (a humanized pan anti-TGF-β mAb): Greco et al. [426] investigated TGF-β upregulation as a mechanism of immune evasion in cancer patients refractory to anti–PD-1/PD-L1 therapies. Analyzing 10,000 patient tumors, the authors revealed a significant correlation between high TGF-β pathway activation and resistance to PD-1/PD-L1 blockade, consistent with TGF-β’s role in suppressing antitumor T cell responses. The study used SAR439459 to demonstrate its ability, in preclinical models with syngeneic mice, to suppress tumor growth using a single agent and to enhance the efficacy of PD-1 blockade by reversing TGF-β-mediated immune suppression and leading to complete and sustained tumor regression. The study provided a compelling foundation for exploring SAR439459’s potential in cancer immunotherapy, urging future research to substantiate its clinical applicability and therapeutic benefits. 

A first-in-human clinical trial (NCT03192345) [427] investigated the safety, tolerability, and preliminary clinical outcomes of SAR439459 either alone or in combination with the PD-1 mAb Cemiplimab, in adult patients with advanced solid tumors. The study included two parts, with dose escalation in Part 1A and combination therapy in Part 1B. As of 31 January 2020, a total of 52 patients were enrolled, and while dose-limiting toxicities were reported, the MTD was not achieved. The treatment led to a reduction in total plasma TGF-β1 levels and induced immune cell activation, suggesting potential therapeutic effects. Although some adverse events were reported, the overall tolerability profile was deemed acceptable. Preliminary results from tumor biopsies indicated inhibition of the TGF-β signaling pathway and a shift in the tumor-immune phenotype. Further dose expansion cohorts are ongoing. Overall, SAR439459 ± Cemiplimab appears promising, but further investigation is needed to assess its efficacy and safety in a larger patient population.

XPA-42-089, a pan-specific anti-TGF-β mAb was tested in a study by Dodagatta-Marristudy et al. [428] to address the challenges of low response rates in checkpoint blockade immunotherapy for metastatic cancer patients, aiming to identify mechanisms to overcome resistance. Using a panel of murine syngeneic squamous cell carcinoma lines, the researchers investigated responses to anti-PD-1, XPA-42-089, and their combination. While anti-PD-1 therapy showed limited efficacy in achieving a complete regression of tumors, it inhibited tumor growth in lines with higher mutation loads. XPA-42-089 monotherapy demonstrated 20% and 10% complete regression for two specific tumor lines, along with the induction of long-term anti-tumor immunity. Combinatorial therapy with α-PD-1 and XPA-42-089 resulted in a synergistic increase in complete regression rates. The study highlights the competing TGF-β-driven immunosuppressive program induced by XPA-42-089 and suggests new opportunities for combinatorial treatment, particularly in SCCs with high mutation loads, CD4+ T cell content, and Phospho-Smad3 signaling. Clinical trials are warranted to validate the potential of α-TGFβ/α-PD-1 combination therapy in human SCC.

Pan-TGFβ mAb, a pan-TGF-β neutralizing mAb developed at Genentech, was tested in toxicology studies in mice and cynomolgus monkeys [102]. This study revealed significant on-target adverse toxicities, notably systemic bleeding, and cardiovascular effects, after 5 weekly intravenous administrations of 30 or 100 mg/kg of Pan-TGFβ mAb followed by a month of recovery. Other drug-related toxicities included histological changes in skin, teeth, tongue, and bone. The authors suggested that the toxicity profile of this biologic was influenced by its binding affinity and potency toward all three TGF-β isoforms. The study emphasized the need for a comprehensive investigation into the specific roles of blocking individual isoforms of TGF-β in cardiovascular toxicity and highlighted the importance of understanding these interactions in developing safe and effective TGF-β pathway inhibitors.

IMC-TR1 (LY3022859) is a TβRII mAb that has shown promising responses in mouse models of breast and colon cancer. This drug was moved to a multi-center, nonrandomized, dose-escalation phase I trial to test its safety in 14 patients with standard therapy-resistant advanced solid tumors (NCT01646203) [429]. Patients were infused with 12.5 or 25 mg IMC-TRI once every two weeks. Significant safety concerns, including cytokine release syndrome and infusion-related reactions, hindered the determination of an MTD. Despite protocol amendments, such as introducing prophylactic therapy and adjusting the dosing regimen, infusion-related reactions persisted, particularly at the 25 mg dose level. Challenges in obtaining a pharmacokinetic profile at the initial dose level and subsequent profiles indicating insufficient exposure made it impractical to achieve target trough levels for efficacy. The study discussed two potential etiologies for infusion reactions: an off-target activity of the antibody and binding of the drug to most host cells which are positive for TβRII. Ultimately, the primary objective of determining a safe dose without infusion-related reactions at biologically active levels was not met.

Anti-LAP: TGF-β1 LAP is expressed on multiple immune cells, is overexpressed in tumors, and predicts poor outcomes [430]. Gabriely et al. [430] developed two new anti-LAP mouse mAbs (against TGF-β1 LAP) and tested their effect on antitumor immune responses in syngeneic mouse models of melanoma, colorectal carcinoma, and GBM. The investigators showed that anti-LAP (10 mg/kg every 3 days) decreased tumor growth, decreased LAP+ Tregs and tolerogenic dendritic cells, and blocked TGF-β release. They also identified a role for CD103+ CD8 T cells in cancer, characterizing them as having a tolerogenic phenotype. Anti-LAP was shown to modulate DC subsets, enhance antitumor adaptive immune response, and affect tolerogenic CD103+ CD8 T cells. The combination of anti-LAP treatment with antigen-specific vaccination improved tumor immunotherapy and enhanced immune memory. Overall, this study suggests that anti-LAP targets multiple immunoregulatory pathways and could be an effective immunotherapeutic. However, this study did not characterize the specificity of anti-LAP on TGF-β isoforms.

SRK-181 (anti-latent TGF-β1 mAb): Despite its enormous success, many cancer patients fail to respond to anti-PD-1 immune checkpoint blockade therapy owing to intrinsic or acquired resistance [468]. Evidence supports that the immunosuppressive action of TGF-β signaling plays a role in mediating resistance to anti-PD-1 therapy [469,470,471]. To counteract the effect of TGF-β signaling on resistance to cancer checkpoint PD-1 blockade therapy, Martin et al. [64] developed a high-affinity, humanized antibody named SRK-181 that specifically neutralizes latent TGF-β1, but not the other latent TGF-β isoforms and exclusively inhibits the activation of only the TGF-β1 isoform. SRK-181 was tested in syngeneic mouse cancer models (urothelial cancer, melanoma, and breast cancer) resistant to anti-PD-1 treatment. The coadministration of SRK-181 and anti–PD-1 mAb induced robust and synergistic antitumor responses, increased intratumoral CD8+ T cells, decreased immunosuppressive myeloid cells, and improved survival of syngeneic mice bearing tumors resistant to anti-PD-1. This selective TGF-β1 inhibition showed effectiveness even in tumors expressing multiple TGF-β isoforms, without observed cardiotoxicities in animal studies found with pan-TGF-β blockade. The findings propose selective TGF-β1 inhibition as a promising approach to overcome primary resistance to immune checkpoint blockade therapy. 

To pave the way for clinical development, Welsh et al. [431] conducted a thorough preclinical evaluation of SRK-181′s pharmacokinetics, pharmacodynamics, and safety in rodents and monkeys. Four-week toxicology studies reveal that weekly intravenous administration of SRK-181 was well-tolerated with sustained plasma levels of this mAb in rats and monkeys without treatment-related adverse effects at the highest doses tested, 200 mg/kg in rats and 300 mg/kg in monkeys. There were no drug-related adverse effects even 4 weeks after treatment was stopped. These studies support SRK-181′s substantial efficacy and broad therapeutic window, compelling its use in a first-in-human, multi-center, open-label, Phase 1 trial (NCT04291079), which is currently active (last updated 1 November 2024). This trial aims to assess the safety, tolerability, pharmacodynamics, pharmacokinetics, and efficacy of SRK-181 in adult patients with locally advanced or metastatic solid tumors. The investigation will involve dose escalation and expansion, and the administration of SRK-181 will be studied both as a standalone treatment and in combination with anti-PD-(L)1 therapy. 

ABBV-151, PIIO-1, and DS-1055a (human anti-GARP mAbs): Glycoprotein-A repetitions predominant (GARP), a cell surface protein present on certain hematopoietic cells such as platelets, Tregs, and B lymphocytes, plays a role in immune tolerance through activating latent TGF-β1 [127]. GARP is also enriched in many cancers. A phase I clinical trial (NCT03821935) [432] investigated the impact of blocking TGF-β activation by GARP using ABBV-151, an anti-GARP mAb, on cancer immunotherapy in combination with the anti-PD-1 MoAb Budigalimab. Of the 248 patients with locally advanced or metastatic solid tumors enrolled in the study, patients received either ABBV-151 as monotherapy or in combination with Budigalimab. ABBV-151 as a single agent or with Budigalimab was well-tolerated and significantly enhanced response in some patient subgroups, particularly those with urothelial cancer relapsed/refractory to PD-1 inhibition. However, the overall objective response rate (ORR) in the entire population was 10%, with varying response rates in different tumor types. Safety concerns were noted, with 17% of patients experiencing grade 3 or greater adverse events related to the study drugs, leading to treatment discontinuation in 12% of patients. The hepatocellular carcinoma (HCC) cohort was paused due to safety concerns, ultimately leading to its dissolution. While the study provides insights into the potential benefits of the combination therapy, safety issues, especially in the HCC cohort, raise concerns about the overall risk–benefit profile. Following up with this, in a study utilizing a large database, investigators showed that cancers that have an overexpression of GARP are resistant to immune checkpoint blockade [407]. They demonstrated treatment with anti-GARP mAb (PIIO-1) showed effectiveness in murine cancer models by preventing thrombocytopenia, preferentially accumulating in the TME, and enhancing CD8+ T cell function while reducing TGF-β signaling. The study concluded that GARP contributes to immune resistance in cancer and proposes PIIO-1 as a promising immunotherapeutic strategy to overcome primary resistance to anti-PD-1. While the research adds valuable insights, further validation in clinical trials and the exploration of potential limitations are crucial for establishing the generalizability of the findings across diverse cancer types and understanding the long-term effects of PIIO-1. A different group of investigators developed another human anti-GARP mAb (DS-1055a) that robustly blocked GARP in the TME and suppressed the growth of HT-29 human tumors in humanized mice [433]. 

C6D4 (mouse anti-αVβ8 mAb): As discussed earlier in this review, certain integrins that are overexpressed in tumor cells, such as αVβ6 and αVβ8, activate latent TGF-βs 1 and 3. This makes those integrins attractive targets for TGF-β blockade therapies. Takashaka and colleagues [434] showed that the expression of αvβ8 by tumor cells plays a crucial role in driving tumor growth in vivo, and the blockade of αvβ8 with the mouse MAb C6D4 (10 mg/kg, once to twice weekly) can significantly reduce tumor growth and improve survival. The findings suggest that αvβ8-expressing tumor cells serve as a platform for TGF-β activation, influencing the complex interaction network between tumor cells and immune cells. The investigators proposed targeting tumors with high β8 expression using neutralizing αvβ8 antibodies, either alone or in combination with PD-1/PD-L1 inhibitors, as a potential therapeutic approach. 

ADWA-11 (mouse anti-αVβ8 mAb): In an impressive study published in Cell Reports in 2021, a team of researchers who developed a new mouse mAb designed to block αvβ8 (named ADWA-11), demonstrated its significant efficacy in suppressing or completely regressing the growth of squamous cell carcinoma, mammary cancer, colon cancer, and prostate cancer in syngeneic models [435]. This effect was particularly pronounced when ADWA-11 was combined with other immunomodulators or radiotherapy. The expression of αvβ8 was highest in CD4+CD25+ T cells within tumors and deleting β8 specifically from T cells proved as effective as ADWA-11 in inhibiting tumor growth. ADWA-11 also enhanced the expression of genes associated with tumor cell killing in CD8+ T cells, counteracting the inhibitory effects of TGF-β. The study underscored αvβ8 integrin as a promising target for cancer immunotherapy, emphasizing its potential to induce tumor regression and fostering durable anti-tumor immunity.

### 12.8. Ligand Traps

This category includes TGF-β fusion proteins such as Fc-TβRII and Fc-TβRIII. These agents are generated using a molecular biology approach of fusing the Fc region of human IgG with the cytoplasmic domains of TβRII and TβRIII. The Fc increases the stability and hence the half-life of those traps to that comparable to IgGs, 21–28 days. These agents can be systemically administered or injected directly into tumors. Fc-TβRII selectively neutralizes TGF-β1 and 3, while Fc-TβRIII effectively traps all TGF-β isoforms and related proteins. Studies using these traps have demonstrated efficacy against metastatic tumor growth in mice. Additionally, the viral delivery of Fc-TβRII has shown promise in enhancing tumor-reactive CD8+ T cell responses, potentially aiding in prostate cancer vaccine development. 

AVID200 (Fc-TβRII): A phase Ib clinical trial (NCT03895112) [436] explored the safety and therapeutic potential of AVID200 (Fc-TβRII), which traps TGF-β1 and TGF-β3, for myelofibrosis (MF), a clonal myeloproliferative neoplasm. In a cohort of 21 patients with advanced MF, AVID200 showed no dose-limiting toxicity, with grade 3/4 anemia and thrombocytopenia occurring in a subset of patients. Two patients achieved clinical benefit, and spleen and symptom improvements were observed. Notably, platelet counts increased in 81% of treated patients, suggesting a positive impact on thrombocytopenia. AVID200 also effectively suppressed plasma TGF-β1 levels and phospho-Smad2^S465/467^ in MF cells. The study concludes that AVID200 is well-tolerated and holds promise as a rational therapeutic option for MF, particularly in combination with agents targeting aberrant MF intracellular signaling pathways, warranting further evaluation.

### 12.9. Bifunctional Fusion Proteins

Bifunctional fusion proteins are genetically engineered molecules composed of two distinct functional domains—a targeting or binding domain and an effector domain—combined for therapeutic purposes. These proteins offer targeted therapy by precisely localizing to specific cells, enhancing specificity, and minimizing off-target effects. Their multimodal action, often designed for immunotherapy applications, allows for the simultaneous engagement of different therapeutic mechanisms. The customizable design of bifunctional fusion proteins enables researchers to tailor molecules to specific diseases, reducing side effects associated with traditional therapies. With versatility across various therapeutic areas, these proteins exemplify a promising approach in biotechnology for developing precise and effective treatments. 

4T-Trap is a fusion protein of the TGF-β binding domain of TβRII to human anti-CD4 IgG, which was made to selectively suppress TGF-β signaling in CD4+ lymph nodes rather than globally [437]. Twice weekly intravenous injections of tumor-bearing mice with 0.1 mg 4T-Trap selectively inhibited Th cell TGF-β signaling in tumor-draining lymph nodes, leading to an IL-4-dependent tumor vasculature reorganization and cancer cell death in four weeks. Additionally, 4T-Trap induced tumor hypoxia, resulting in increased VEGFA expression. Combined VEGF inhibition with 4T-Trap enhanced starvation-triggered cancer cell death and amplified the anti-tumor effect. The findings suggest that targeted TGF-β signaling blockade in helper T cells can trigger an effective tissue-level cancer defense response, supporting cancer environment-directed therapies.

Bintrafusp Alfa (M7824). Bintrafusp Alfa is a new bifunctional fusion protein comprising the extracellular TβRII (a TGF-β Trap) anchored to the Fab region of human anti-PD-1 mAb [438]. The bifunctionality of Bintrafusp Alfa was designed to allow a more targeted approach to blocking TGF-β activity in tumors expressing higher PD-1 compared to host tissue, thereby potentially reducing host toxicity compared with using the combined treatment of those inhibitors in separate molecules. In preclinical investigations, Bintrafusp Alfa demonstrated extended survival and conferred long-term protective immunity when compared to TGF-β blockade or anti-PD-L1 antibody alone [438]. M7824 exhibited a reduction in regulatory T-cell function, a substantial increase in CD8+ T-cell and natural killer cell infiltration, and a decrease in myeloid-derived suppressor cell infiltration within tumors [319,472,473]. The safety and effectiveness of Bintrafusp Alfa were first investigated in patients with advanced NSCLC [440]. The study, part of an ongoing phase 1 trial (NCT02517398), included 80 patients who had experienced disease progression after platinum doublet treatment or platinum-based adjuvant/neoadjuvant therapy and with no prior immunotherapy. Patients were randomly assigned to receive either a 500 mg or 1200 mg dose of Bintrafusp Alfa every 2 weeks. The primary endpoint, assessed by the ORR, revealed an overall ORR of 21.3%, with the 1200 mg dose demonstrating a higher ORR (25.0%) compared to the 500 mg dose (17.5%). Notably, patients with PD-L1-positive and PD-L1-high expression exhibited higher response rates. Treatment-related adverse events occurred in 69% of patients, with 29% experiencing grade 3 or higher events. Although 10% of patients discontinued treatment due to adverse events, there were no treatment-related deaths. The overall safety was similar to established checkpoint inhibitors. The study supported further trials of Bintrafusp Alfa in various NSCLC treatment settings. 

The efficacy and safety of Bintrafusp Alfa were further studied in Asian patients with advanced gastric/gastroesophageal junction cancer who had limited treatment options after first-line therapy [441]. The study, conducted as an expansion cohort of an ongoing phase I trial (NCT02699515), included 31 heavily pretreated patients who received 1200 mg Bintrafusp Alfa via intravenous infusion over 1 h once every 2 weeks until disease progression, unacceptable toxicity, or withdrawal. The primary objective was to assess safety and tolerability. Tumor regression was assessed every 6 weeks by CT or MRI, and confirmed by blinded assessment with other scans. Results showed a manageable safety profile, with 19% experiencing grade 3 treatment-related adverse events and a 16% confirmed objective response rate. The median duration of response was 8.7 months, and responses occurred regardless of PD-L1 positivity or microsatellite instability, but correlating with high tumor TGF-β levels. The study suggests that Bintrafusp Alfa has potential clinical activity in this patient population.

Many other ongoing and completed clinical trials investigating Bintrafusp Alfa in different types of cancer include hepatocellular carcinoma [442], squamous cell carcinoma of the head and neck [443], human papillomavirus-associated malignancies [474], esophageal squamous cell carcinoma [475], esophageal adenocarcinoma [476], CRC [477], and biliary tract cancer [478]. These trials demonstrated signs of clinical efficacy and manageable safety profiles in various patient populations, providing evidence for a further investigation of Bintrafusp Alfa in advanced cancers. 

A population pharmacokinetic analysis of Bintrafusp Alfa compiled from 644 patients with various solid tumors estimated a mean elimination half-life of 6.93 days (95% CI 4.69–9.65 days) [479]. This is in contrast to the mean elimination half-life of 27 days for Pembrolizumab [480], typical for anti-PD-1 mAbs. Recently, a phase 3 trial was conducted on a specific population of patients with high PD-L1–expressing advanced NSCLC (NCT03631706) [444]. However, the study did not achieve its primary endpoint of superior progression-free survival (PFS) with Bintrafusp Alfa, not attaining a significant efficacy benefit over pembrolizumab in first-line treatment, as evidenced by a median PFS of 7.0 months compared to 11.1 months with Pembrolizumab. The study was discontinued prematurely, and despite similar OS in exploratory analysis, efficacy findings contradicted earlier studies with Bintrafusp Alfa. Higher adverse event rates were observed in the Bintrafusp Alfa arm, with specific side effects such as bleeding and anemia being more common. The study suggests that the pleiotropic nature of TGF-β signaling, potential drug resistance, and a need for further patient selection beyond PD-L1 status may impact the efficacy of dual-targeted immunotherapies like Bintrafusp Alfa in NSCLC. The limitations include the open-label design and a shorter treatment interval for Bintrafusp Alfa compared to Pembrolizumab, emphasizing the need for additional investigation to determine optimal treatment sequences, combinations, and patient populations for TGF-β blockade. It is also possible that Bintrafusp Alfa has more off-target effects compared to Pembrolizumab.

A preclinical study with radiolabeled-Bintrafusp Alfa showed that while this compound builds up in tumors, significant amounts build up in the heart, lung, spleen, liver, and bone of tumor-bearing mice [439]. A PET imaging study of 89Zr-Pembrolizumab showed low uptake in the brain, lung, bone cortex, subcutaneous tissue, and abdominal cavity, moderate uptake in liver and kidney, and high uptake in tumor, spleen, and bone marrow [481]. The tumor uptake of 89Zr-Pembrolizumab correlated with treatment response and survival. Based on this, Pembrolizumab may have less off-target effects than Bintrafusp Alfa, possibly explaining the results of the phase 3 trial with Bintrafusp Alfa.

SHR-1701, a bifunctional anti-PD-L1/TGF-βRII fusion protein consisting of the Fab domain of human anti-PD-L1 fused to the extracellular domain of human TβRII, underwent a phase I study (NCT03774979) [482] targeting 32 patients with recurrent or metastatic cervical cancer post-platinum treatment. The results indicated an ORR of 15.6%, with an ongoing response observed in 80.0% of responders. The disease control rate was 50.0%, and the 6-month duration of response rate stood at 80.0%. Median PFS was reported as 2.7 months, but when evaluated by immune-modified RECIST, it extended to 4.1 months. The OS rate at 12 months reached 54.6%. Notably, treatment-related adverse events of grade 3 or 4 were documented in 34.4% of patients, with no treatment-related deaths. This study suggests that SHR-1701 exhibits promising antitumor activity and manageable safety, presenting a potential treatment option for recurrent or metastatic cervical cancer following platinum-based regimens. Further exploration of SHR-1701’s therapeutic potential involved a phase 1 trial with 171 patients having pretreated advanced solid tumors (NCT03710265) [483]. During the dose-escalation phase, no dose-limiting toxicity was observed, establishing 30 mg/kg every 3 weeks as the suggested phase 2 dose. In the clinical expansion phase, SHR-1701 showcased promising antitumor activity, particularly in gastric cancer, with a 20.0% ORR and a one-year OS of 54.5%. The study concluded that SHR-1701 maintains an adequate safety profile and holds promising therapeutic potential in advanced solid tumors, paving the way for further investigation.

A preclinical study aimed to identify predictive factors for lung cancer patients resistant to PD-1/PD-L1 inhibitors but responsive to second-generation agents like SHR-1701 [484]. The research utilized multivariable Cox regression to examine the connection between clinical outcomes of PD-1/PD-L1 inhibitor treatment and lymphocyte recovery in lung cancer patients. Poor lymphocyte recovery was found to be linked to shorter PPS, an increase in Tregs, and a decrease in CD8+ T cells in the peripheral blood of patients treated with anti-PD-1/PD-L1 antibodies. Murine models further demonstrated that mice with impaired lymphocyte recovery after chemotherapy showed imbalances in Treg cells and CD8+ T cells in tumors and immune organs. These mice did not respond to anti-PD-1 therapy but stayed sensitive to SHR-1701. The findings suggest that lung cancer patients with poor lymphocyte recovery may be resistant to traditional PD-1/PD-L1 inhibitors but potentially responsive to second-generation agents like SHR-1701.

YM101 is a bispecific antibody of pan-TGF-β and PD-L1, developed using the Check-BODY™ technology platform, that aims to enhance the effectiveness of anti-PD-1/PD-L1 therapies and alleviate drug resistance [445]. The bioactivity of YM101 was validated through various assays, demonstrating its ability to counteract the biological effects of TGF-β and PD-1/PD-L1 pathways. Experiments using EMT-6 (breast cancer), CT26 (colon cancer), and 3LL (murine T cells) tumor syngeneic mouse models showed that YM101 has superior anti-tumor activity compared to anti-TGF-β and anti-PD-L1 monotherapies. Mechanistically, YM101 fostered the creation of a ‘hot tumor’ by modulating the TME, amplifying the numbers of tumor-infiltrating dendritic cells and lymphocytes, bumping up the M1/M2 ratio, and increasing the production of cytokine in T cells. These findings suggest that YM101 could be a promising therapeutic strategy for cancers by simultaneously blocking TGF-β and PD-L1 pathways, leading to a robust anti-tumor effect.

BiTP is a bispecific antibody of pan-TGF-β and PD-L1. Building on the previous pilot study using YM101, Yi et al. [446] recently explored the antitumor effect of BiTP on TNBC using murine models. BiTP exhibited high binding affinity to both targets, effectively counteracted signaling pathways, and demonstrated superior antitumor activity in murine TNBC models compared to anti-PD-L1 and anti-TGF-β monotherapy. BiTP also improved the TME by reducing collagen deposition and enhancing immune cell penetration, suggesting its potential as a promising agent for TNBC treatment.

### 12.10. Antisense Oligonucleotides (ASOs)

Oligonucleotide therapeutics represent a burgeoning class of drugs comprising various modified or unmodified short nucleic acid molecules, including ASOs, small interfering RNA, microRNA, aptamers, and DNAzymes. These therapeutics exert their effects through mechanisms such as gene silencing, steric blocking, or splicing modulation via Watson–Crick base pairing to targeted mRNAs. While oligonucleotide therapeutics have received FDA approval for diverse indications, particularly addressing single gene mutations, such as blocking translation or inducing RNase H-dependent degradation, their application in oncology remains investigational, with numerous clinical trials underway [485]. Compared to traditional small molecules and other targeted therapies, oligonucleotide therapeutics boast simpler design approaches, shorter synthesis times, and lower costs, thanks to their high affinity to targets based on sequence matches. Moreover, their straightforward structure and reproducible chemistry ensure known safety profiles, making them suitable for combination therapies [485]. However, challenges persist, including optimizing drug delivery efficiency through advancements in nucleic acid chemistry and delivery modes [485].

ASOs are short strands of DNA that bind to specific RNA targets via base pairing. However, their unmodified form is quickly degraded in cells. To address this, chemical modifications are applied to promote cellular uptake and resistance to degradation. These modifications include phosphorothioate (PS) backbones, methyl-phosphonate (MP), and N3′-P5′ phosphoramidate (NP) substitutions, and sugar modifications like 2′-O-methyl (2′-OMe) and 2′-O-methoxyethyl (2′-MOE) [485]. Second- and third-generation ASOs incorporate these modifications to increase target binding affinity and reduce toxicity. Further advancements involve locked nucleic acids (LNAs), constrained methoxyethyl (cMOE), phosphorodiamidate morpholino oligomers (PMOs), and peptide nucleic acids (PNAs), each offering specific benefits in stability, affinity, and toxicity profiles [485]. Additionally, ASOs can be conjugated with various molecules for targeted delivery or enhanced cellular uptake.

Trabedersen (AP12009), a TGF-β2 phosphorothioate-modified ASO, has been used as an intratumoral injectable in clinical trials for testing its safety and the tolerability of aggressive tumors shown to express high levels of TGF-β2 mRNA (in colorectal neoplasm, melanoma, PC) (NCT00844064), for efficacy and safety in patients with recurrent or refractory anaplastic astrocytoma or secondary GBM (NCT00431561, NCT00761280) [450], and in combination with Atezolizumab for the treatment of metastatic or recurrent NSCLC (NCT05935774, study withdrawn). Extending from evidence that aggressive gliomas express high levels of TGF-β2 believed to contribute to disease progression, the above phase IIb study was conducted to compare the safety and effectiveness of Trabedersen delivered directly into tumors with standard chemotherapy in patients with recurrent/refractory high-grade glioma [450]. A total of 145 patients with recurrent/refractory GBM multiforme or anaplastic astrocytoma were arbitrarily allotted to receive either one or two doses of Trabedersen or standard chemotherapy. The primary endpoint was 6-month tumor growth, with secondary endpoints including response rates at different time points, survival outcomes, and safety. Despite no significant improvement in the primary endpoint, Trabedersen demonstrated promising survival outcomes in a small subgroup of patients. However, concerns arise regarding the trial’s methodology, including differences in patient characteristics, inadequacies in the chemotherapy regimen, flawed analysis methods, and endpoint discrepancies [486]. No other cancer clinical trial is currently active for Trabedersen. The failure of Trebedersen in clinical trials could be due to the use of TGF-β1 instead of TGF-β2 as the more appropriate target, supported by Kaplan–Meier and multivariant analysis, where levels of TGF-β1 mRNA were significantly elevated over those of TGF-β2, and TGF-β1 levels were better correlated with poor OS and progression-free survival [487]. In addition, the over-reliance on TGF-β mRNAs as a readout of TGF-β isoform activity is weak, given that mRNA level comparisons by RT-PCR were assessed as relative to normal tissue and thus do not reflect the absolute amount of message. Moreover, there could be differences in the translation of those TGF-β isoforms, differences in their activation as well as differences in their biological activities, given that α2M preferentially neutralizes TGF-β2, and also that TGF-β2 but not TGF-β1 signaling requires β-glycan (Figure 2 and Figure 3). 

Trabedersen was also tested in a preclinical model of PC [488]. Although these investigators reported encouraging results, such as the suppression of TGF-β2 expression and suppression of tumor growth, the investigators did not use a suitable ASO control to confirm that the observed tumor and immune response was not triggered by the modified oligonucleotide. 

ISTH0036 is another TGF-β2 ASO studied in a first-in-human clinical trial for safety and tolerability in patients with glaucoma following intravitreal injection of ISTH0036 (NCT02406833) [452]. An intravitreal injection of ISTH0036 in these patients was safe and likely effective, although the small patient pool of the study was too low to yield statistically significant results.

AP11014 is a TGF-β1 ASO used in preclinical models of prostate cancer, CRC, and NSCLC, with encouraging results [453]. However, results from this study were reported as a meeting abstract in 2004, without a formal publication to date. 

ISTH0047 and ISTH10047: Papachristodoulou et al. [454] explored the efficacy of two new phosphorothioate-locked nucleic acid (LNA)-modified ASO gapmers, ISTH1047 and ISTH0047, designed to specifically inhibit TGF-β1 and TGF-β2, respectively. They showed that these ASOs effectively suppressed the expression of the targeted TGF-β isoforms, disrupted downstream signaling pathways, and hindered the growth and invasiveness of glioma cells. Importantly, the systemic administration of these oligonucleotides in glioma-bearing mice led to prolonged survival and reduced tumor progression, suggesting their potential as a promising therapeutic strategy for glioma treatment in human patients. However, it appears the investigators did not use a nonspecific modified oligonucleotide control in their studies, reducing the strength of their studies. 

### 12.11. Aptamers

**Peptide Aptamers** are innovative combinatorial proteins, typically consisting of short amino acid sequences (5–20 residues) embedded within a stable protein scaffold. Originating from the concept of Antikörper (Antibodies), peptide aptamers offer an attractive alternative to antibodies in biomedical applications [489]. Unlike antibodies, peptide aptamers are smaller, less immunogenic, and can be rapidly generated in vitro. They are particularly advantageous for interrogating intracellular targets due to their small size and stability. The “loop on a frame” design, proposed by Roger Brent, involves a short peptide loop grafted onto a stable protein backbone, allowing for the selection of high-affinity binders. These innovative molecules, along with other engineered scaffolds, present promising alternatives to traditional antibodies, overcoming limitations in terms of size, production, and adaptability to diverse target surfaces in various biomedical applications. Peptide aptamers thus offer a unique approach to selectively target the oncogenic aspects of TGF-β signaling while preserving its tumor-suppressive effects. To date, they have been underexplored for TGF-β blockade therapeutics. 

Trx-SARA is a peptide aptamer designed to specifically disrupt Smad-dependent TGF-β signaling. In contrast to the commonly used inhibitory Smad, Smad7, Trx-SARA selectively binds to Smad2 and Smad3, inhibiting TGF-β-induced gene responses and EMT in NMuMG murine mammary epithelial cells [447]. Notably, Trx-SARA did not impact the phosphorylation levels of Smad2 or Smad3 induced by TGF-β1. Trx-SARA primarily localized to the nucleus, altering the normal cytoplasmic localization of Smad2 and Smad3, reducing their complex formation with Smad4 after TGF-β1 stimulation. This disruption of active Smad complexes suggests a distinct mode of action for Trx-SARA compared to Smad7. The findings underscore Trx-SARA’s potential as a tool for the targeted manipulation of Smad-dependent signaling pathways, offering insights into molecular mechanisms and paving the way for further exploration of its applications and limitations in diverse cellular contexts and physiological conditions. However, due to its large size and properties, Trx-SARA was introduced in cells with a replicative incompetent retrovirus. 

**Nucleic Acid Aptamers**: Nucleic acid aptamers, short DNA or RNA strands chosen for their target binding affinity, were developed in 1990 through the Systematic Evolution of Ligands by Exponential Enrichment (SELEX) method [447]. Like antibodies, nucleic acid aptamers adopt unique three-dimensional structures for precise molecular recognition. Despite challenges in clinical development, including physicochemical characteristics and production costs, recent progress in nucleic acid aptamer selection and formulation, guided by lessons from nucleic acid clinical development, has encouraged numerous investigators to pursue therapeutic aptamers.

APT-β1: To selectively target the TGF-β1 isoform in cancer, Takahashi et al. [448], using the SELEX method, recently developed a high affinity and specificity RNA aptamer (named APT-β1) targeting active human TGF-β1 but not TGF-βs 2 and 3. Picomolar concentrations of APT-β1 demonstrated potent inhibition of TGF-β1-induced signaling and cell morphology in both in vitro and in vivo studies. When administered alone in mice bearing a NSCLC xenograft, APT-β1 (10 mg/kg/day s.c. for 3 weeks) exhibited minimal impact on tumor growth, prompting scrutiny of its standalone efficacy. Intriguingly, APT-β1 significantly enhanced the anti-tumor effect of Gefitinib (100 mg/kg/day, p.o.), a tyrosine kinase inhibitor targeting mutant EGFR, suggesting potential synergy in combination therapies to combat lung cancer. Further exploration is needed to understand the nuanced interplay between APT-β1 and other therapeutic agents for effective cancer treatment. While APT-β1 exhibited much higher potency compared to anti-TGF-β1 mAb, its pharmacokinetics are poor compared to antibody therapies. However, the half-life of aptamers has been improved by conjugation with molecules that reduce renal clearance [447].

Aptamer S58: Zhu et al. [449] aimed to identify aptamers binding to the extracellular segment of TβRII and assess their impact on TGF-β-induced transdifferentiation of fibroblasts. Employing SELEX from a single-stranded DNA library, they obtained twenty-one sequences after eight rounds of selection. They then isolated two key sequences, aptamers S58 and S68, for further investigation. Aptamers S58 (20 nM and 100 nM), but not S68, demonstrated a significant inhibitory effect on α-smooth muscle actin expression and its incorporation into actin stress fibers induced by 2 ng/mL of TGF-β2. Additionally, the same concentrations of S58, but not S68, suppressed TGF-β2-induced cell contraction and inhibited the phosphorylation and nuclear translocation of Smad2. These findings suggest that aptamer S58 has potential therapeutic implications in modulating TGF-β-induced fibroblast transdifferentiation. 

## 13. Summary and Future Prospects

TGF-βs have emerged as promising therapeutic targets for cancer and fibrosis. Over the past 15 years, numerous drugs have been developed, tested in animal models, and brought into clinical trials with promising yet mixed results. Although some of these drugs have shown encouraging tumor response with limited host toxicity, others showed no significant response or unacceptable adverse reactions. 

These toxicities seemed to be associated with abrogating the homeostatic functions of TGF-βs. Strategies to reduce adverse effects while maintaining TGF-βs’ important roles in normal tissues include reducing drug doses to work within therapeutic windows and employing intermittent drug treatment. 

TGF-β isoform knockout studies show each isoform is involved in unique developmental and physiological functions, with TGF-β1 being most important in immune regulation. Given TGF-β1’s role in promoting tumor growth by suppressing tumor immune surveillance, selective antagonism of TGF-β1 is expected to hold therapeutic promise. However, the TGF-β1-selective monoclonal antibody TβM1 did not generate a useful tumor response compared to pan-TGF-β inhibitors, likely due to its low affinity or poor tumor penetration [374]. In contrast, the mAb SRK-181, which blocks TGF-β1 LAP, generated impressive robust tumor responses (in combination with anti-PD-1) without noticeable adverse effects. The overall results of many preclinical mouse studies support that TGF-β1-selective antagonists have significant tumor response with good tolerability and a low chance of serious adverse effects. However, rigorous clinical studies are necessary before their true value in patients can be adequately assessed. 

A key challenge is the heterogeneous nature of cancers, with various tumors exhibiting different defects in the TGF-β pathway. Monitoring these defects via liquid biopsies is expected to improve the implementation of appropriate TGF-β blockade strategies. Importantly, TGF-β blockade therapies have shown the greatest therapeutic benefit when used in combination with checkpoint blockade and chemotherapies. This can be explained by the ability of TGF-βs to mediate resistance to both forms of therapy. 

Chemotherapeutic drugs, which promote growth arrest and apoptosis, may switch the TGF-β isoform from TGF-β1 to TGF-βs 2 and 3, consistent with stimuli driving their respective expression patterns (Figure 1). If so, TGF-β2 and TGF-β3 dual blockade or pan-TGF-β blockade may be more effective in suppressing the generation of minimal residual disease than TGF-β1-selective blockade. In contrast, TGF-β1 isoform blockade may work best in combination therapy with anti-PD-1 or anti-PD-L1 agents due to robust immunosuppression by the TGF-β1 isoform. These possibilities underscore the importance of monitoring the expression of TGF-β isoforms in cancers before and continuously during therapy to implement the most effective TGF-β inhibitor. 

Ideally, strategies selectively targeting TGF-β in the TME rather than systemically would offer the most therapeutic benefit while limiting toxicity. Bifunctional proteins hold promise for selectively delivering TGF-β blockade to tumors. These target TGF-β activation in tumors by either (1) GARP inhibitors (i.e., ABBV-151, PIIO-1, and DS-1055a) or (2) integrin αVβ8 antagonists (C6D4 and ADWA-11). The bifunctional proteins, particularly the TGF-β ligand Trap-anti-PD-1 mAb (i.e., Bintrafusp Alfa and SHR1701), designed to selectively neutralize TGF-βs 1 and 3 in tumors (targeted to tumors with the anti-PD-1 moiety) gave impressive tumor responses with acceptable safety profiles compared to standard checkpoint therapies. Other bifunctional agents developed (YM101 and BiTP) also showed good tumor response in preclinical studies. Understanding the extent and type of non-canonical TGF-β pathway activation in a particular malignancy could enable additional therapeutic opportunities through combination therapies.

In conclusion, while TGF-β inhibitors hold significant promise for cancer therapy, there remain challenges to be addressed. Future research efforts should focus on (1) developing a more thorough understanding of TGF-β isoform regulation of expression and activation in cancer, (2) developing more selective TGF-β isoform-specific inhibitors, and (3) refining patient stratification strategies to optimize the efficacy of TGF-β targeted therapies.

## Data Availability

Data sharing is not applicable.

## Abbreviations

**ALK1, ALK2, ALK3, ALK4, ALK5, ALK6 and ALK7**: activin-receptor-like kinase 1, 2, 3, 4, 5, 6, and 7, respectively; **Akt**: Akt/PKB serine-threonine kinase; **α2M**: α2-macroglobulin; **AMPK**: adenosine monophosphate kinase; **AP-1**: activation protein-1; **ASO**: antisense oligonucleotide; **ATF1, ATF2**: activating transcription factor 1 and 2, respectively; **AZGP1**: zinc-alpha2-glycoprotein; **BAMBI**: BMP, activin, membrane-bound inhibitor; **Bcl-2**: B-cell lymphoma-2 protein; **Bcl-xl**: Bcl-2-like gene 1- extra-large; **BDNF**: brain-derived neurotrophic factor; **BMP**: bone morphogenetic protein; **CAF**: carcinoma-associated fibroblast; **CBP**: CREB binding protein; **CD4**, **CD8**, **CD44**, and **CD24**: cluster of differentiation 4, 8, 44, and 24, respectively; **CDK**: cyclin-dependent kinase; CRC: colorectal cancer; **CSCs**: cancer stem cells; **CT**: computerized tomography scan; **c-Jun**: cellular Jun transcription factor, subunit of the AP-1 complex; **COVID-19**: coronavirus disease 2019; **cPML**: cytoplasmic promyelocytic leukemia protein; **CREB-1**: cAMP-responsive element binding protein-1; **CREBH**: cAMP-responsive element-binding hepatocyte protein; **DAB2**: disabled-2; **DAXX**: death domain associated protein; **ECM**: extracellular matrix; **EGF**: epidermal growth factor; **EGFR**: epidermal growth factor receptor; **EMT**: epithelial-mesenchymal transition; **ERK**: extracellular signal-regulated kinase; **FKBP12**: FK506-bind protein-12 kDa; **FLIP**: FLICE (FADD-like IL-1β–converting enzyme)-inhibitory protein; **FADD**: Fas-associated death domain; **FOXA1, FOXP3**: forehead box A1 and P3, respectively; **GADD45b**: growth arrest and DNA damage-inducible 45b; **GARP**: glycoprotein A repetitions predominant; **GBM**: glioblastoma; **GEF**: GTP exchange factor; **GRB2**: growth factor receptor-bound protein 2; **GSK-3β**: glycogen synthase kinase 3β; **GST**: glutathione S-transferase; **HB-EGF**: heparin-binding EGF-like growth factor; **HDAC**: histone deacetylase; **HIF-1α**: hypoxia-inducible factor 1α; **Hrs/Hgr**: hepatocyte growth factor-regulated tyrosine kinase substrate; **HoxB7**: homeobox B7 protein; **HPV**: hepatitis C virus; **IFN-γ**: interferon γ; **IGF-I**: insulin-like growth factor; **IL-2**: interleukin-2; **JNK**: c-Jun N-terminal kinase; **LAP**: latency-associated protein; **LMWH**: low molecular weight heparin; **LTBP**: large latent TGF-β binding protein; **MAPK**: mitogen-activated protein kinase; **MECs**: mammary epithelial cells; **MEK**: MAPK kinase; **MF**: myelofibrosis; **MH1**, **MH2**: mad-homology domains 1, 2, respectively; **MHC**: major histocompatibility complex; **MMPs**: metalloproteinases; **MRI**: magnetic resonance imaging; **MSG1**: melanocyte specific gene-related gene 1; **MSS**: mechanical shear stress; **MTD**: maximum tolerated dose; **mTORC1**: mammalian target of rapamycin complex 1; **NDRG2**: N-Myc-downstream-regulated gene-2; **NK**: natural killer; **NFAT**: nuclear factor of activated T cells; **NF-κB**: nuclear factor κB; **NLS**: nuclear localization sequence; **NSCLC**: non-small cell lung cancer; **ORR**: objective response rate; **OS**: overall survival; **p130Cas**: Crk-associated substrate, 130 kDa; **PAI-1**: tPA inhibitor 1; **PAR6**: polarity protein 6; **P/CAF**: p300/CBP-associated factor; **PCTA**: PML competitor for TGIF association; **PC**: pancreatic cancer; **PDA**: pancreatic ductal adenocarcinoma; **PDGF**: platelet-derived growth factor; **PD-1**/**PD-L1**: programmed cell death protein 1/programmed cell death ligand 1; **PET**: positron emission tomography; **PHLPP1**: PH domain and leucine-rich repeat protein phosphatase 1; **PHRF1**: PHD and ring finger domains 1; **PI3K**: phosphoinositide 3-kinase; **PKA, PKB, PKC**: protein kinase A, B, and C, respectively; **PML**: promyelocytic leukemia protein; **PPM1A1**: magnesium-dependent protein phosphatase A1; **RAF**: rapidly accelerated fibrosarcoma kinase; **RAS**: rat sarcoma protein; **RAAS**: renin-angiogenin-aldosterone system; **Rb**: retinoblastoma protein; **RFX**: regulatory factor x; **ROS**: reactive oxygen species; **RORγ2**: RAR-related orphan receptor γ2; **RUNX3**: Runt-related transcription factor 3; **R-Smad**: receptor Smad; **SARA**: Smad anchor for receptor activation; **SBE**: Smad-binding element; **SELEX**: systematic evolution of ligands by exponential enrichment; **SH2**: Src-homology 2; **ShcA**: SH2-containing A; **SLC**: small latent complex; **Smad**: *C. elegans sma* mothers against decapentaplegic; **Smurf1**: Smad-specific E3 ubiquitin ligase 1; **SNIP1**: Smad-interacting protein 1; **SOS**: son of sevenless; **Sox4**: SRY-related HMG-box 4; **PSA**: prostate specific antigen; **STAT**: signal transducer and activator of transcription; **SCCs**: squamous cell carcinomas; **TAK1**: TGF-β activated kinase 1; **TβRI**, **TβRII**, **TβRII**: TGF-β receptors type I, II, and III; **TF**: tissue factor; **TILs**: tumor infiltrating immune cells; **Tregs**: T regulatory cells; **TGF-α**: transforming growth factor-α; **TGF-βs**: transforming growth factor-βs; **TGIF**: TG-interacting factor; **TH1, TH2, TH17**: T helper 1, 2, and 17 cells, respectively; **TME**: tumor microenvironment; **TMEPAI**: TGF-β-induced transmembrane prostate androgen-induced protein; **TNBC**: triple-negative breast cancer; **tPA**: tissue plasminogen activator; **TRAF6**: tumor necrosis factor receptor (TNFR)-associated factor 6; **TSP-1**: thrombospondin-1; **VD**: (1,25-dihdroxyvitamin D_3_).
